# Impact of Multiplex PCR Blood-Culture Identification Panels on Clinical Outcomes, Antimicrobial Stewardship, and Economic Impact in US Hospitals: A Systematic Review and Meta-Analysis

**DOI:** 10.1093/ofid/ofag370

**Published:** 2026-06-25

**Authors:** Jamil Muqtadir, Sameeullah Bhatti, Irshad Batool, Emil P Lesho

**Affiliations:** Department of Infectious Diseases, Rochester General Hospital, Rochester, New York, USA; Department of Critical Care, Mount Sinai Hospital, New York, New York, USA; Department of Gastroenterology, Aga Khan University Hospital, Karachi, Pakistan; Department of Infectious Diseases, Rochester General Hospital, Rochester, New York, USA

**Keywords:** antimicrobial stewardship, bloodstream infection, meta-analysis, multiplex PCR, rapid diagnostics

## Abstract

**Background:**

We performed a systematic review and meta-analysis synthesizing evidence on multiplex PCR blood culture identification (BCID) panels and their effects on clinical, antimicrobial stewardship, and economic outcomes.

**Methods:**

We searched PubMed, Scopus, Embase, CINAHL, Web of Science, and the Cochrane Library for studies evaluating time to appropriate therapy, mortality, length of stay (LOS), antimicrobial optimization, and cost in adult inpatients.

**Results:**

Twenty studies encompassing 4587 patients with bloodstream infections across U.S. hospitals were included. BCID implementation showed shorter time to appropriate antimicrobial therapy by 17.28 hours (95% CI, −24.00 to −10.56) and hospital LOS by 1.25 days (95% CI, −1.79 to −.71). No statistically significant effect on mortality was observed (OR, 1.04; 95% CI, .81–1.34). Economic outcomes were not significantly different.

**Conclusions:**

BCID panels were associated with shorter time to effective therapy; however, a timely clinical response is necessary.

## Teaser

Multiplex PCR blood culture identification panels in US hospitals were associated with earlier appropriate antimicrobial therapy and shorter hospital stay, but not lower mortality. Benefits are likely greatest when rapid results are paired with timely antimicrobial stewardship intervention.

Bloodstream infections are associated with increased risk of death, extended length of hospital stay, and cost [1]. Delays in appropriate therapy, particularly in patients with Gram-negative bacteremia, are associated with worse outcomes [2]. Rapid diagnostic techniques could enhance patient outcomes by enabling more rapid detection of pathogens and reducing the time to appropriate therapy [1].

Conventional blood culture methods require 24–72 hours to identify pathogen(s); therefore, prolonged empirical treatment is required [3]. Multiplex PCR blood culture identification (BCID) panels, including but not limited to, BioFire FilmArray, Verigene, and ePlex, can identify pathogens and resistance markers within 1–8 hours and may decrease the time to appropriate therapy and support antimicrobial stewardship [3]. Evidence on BCID panel impact in US hospitals remains fragmented. This systematic review and meta-analysis quantitatively synthesizes US evidence on BCID panel effects on: (1) time to appropriate therapy, (2) antimicrobial stewardship outcomes, (3) clinical outcomes (mortality, length of stay (LOS)), and (4) economic impact.

## METHODS

This systematic review followed PRISMA guidelines and was registered in PROSPERO (CRD420251232553).

### Search Strategies and Sources of Information

Six databases (PubMed, Scopus, Embase, CINAHL, Web of Science, Cochrane Library) were searched through November 2025 without date restrictions. The complete search strategy is provided in Supplementary Methods 1.

### Eligibility Criteria

Eligibility followed the PICOS framework: hospitalized adults in US settings with positive blood cultures (Population); commercial multiplex PCR BCID panels (Intervention); conventional microbiological methods (Comparator); time to appropriate therapy (primary outcome), with mortality, LOS, antimicrobial optimization, and costs as secondary outcomes (Outcomes); and nonrandomized or randomized comparative designs (Study design). The review was intentionally restricted to U.S.-based studies because BCID implementation, antimicrobial stewardship infrastructure, laboratory reporting workflows, reimbursement models, and hospital cost structures vary substantially across healthcare systems. English-language publications were included to ensure consistent data extraction and interpretation of intervention descriptions, stewardship workflows, and outcome definitions. Studies were excluded if conducted outside the US, in pediatric populations, without commercial BCID panels, or if they were reviews or commentaries.

### Study Selection Process

**Figure 1.**
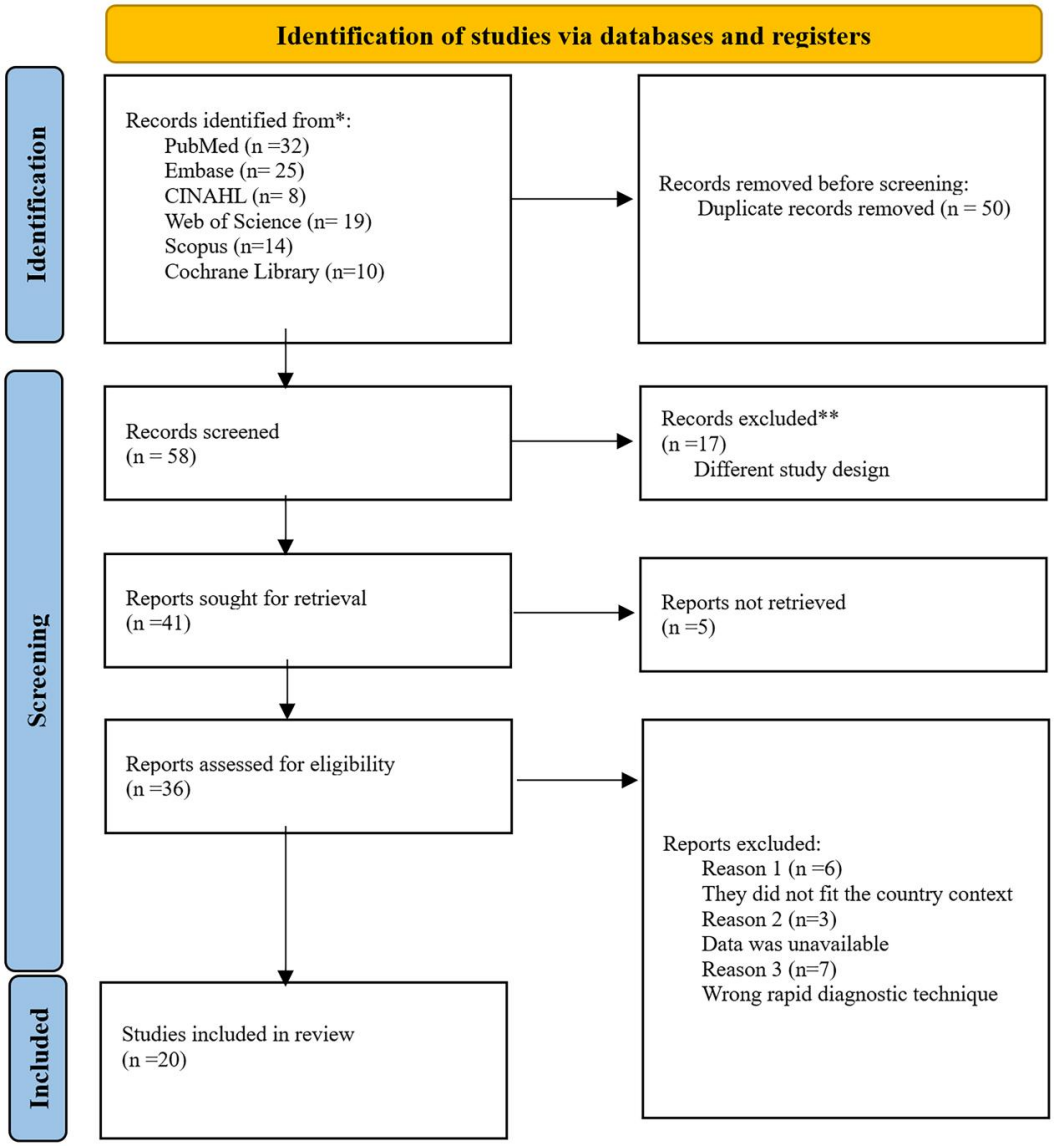
PRISMA flow diagram of study selection. The diagram shows record identification, duplicate removal, screening, full-text assessment, exclusions, and final inclusion of 20 studies evaluating multiplex PCR BCID panels in adult U.S. hospital settings. BCID, blood culture identification; PRISMA, preferred reporting items for systematic reviews and meta-analyses.

Two reviewers independently screened records and assessed full texts. The search identified 108 records; after deduplication (n = 50), screening (n = 17 excluded), and full-text review (5 not retrieved, 16 excluded), 20 studies met inclusion criteria (Figure 1). Studies were published 2013–2024, encompassing 4587 patients. Nineteen employed were nonrandomized designs; one was a randomized trial. Platforms included BioFire FilmArray (n = 8), Verigene (n = 9), and ePlex (n = 3). Eleven studies evaluated BCID with active ASP: 9 assessed BCID with passive reporting. These assays differ in organism targets and resistance-marker menus; Verigene GN-BC and ePlex BCID-GN include Gram-negative resistance markers, including ESBL-associated and carbapenemase genes. Study characteristics are presented in Table 1.

**Table 1.** Key Study and Population Characteristics are Summarized

Author, Year	Design	Sample Size	Demographics	BCID Panel
Donnelly et al 2021 [4]	Retrospective, observational, single-center study	163 patients (98 preintervention and 65 postintervention)	Adults	ePlex BCID
Chiasson et al (2022) [1]	Single-center, pre–post implementation study	180 patients (82 pre, 98 post)	Adults	BioFire FilmArray BCID
Russo HR et al (2019) [5]	Single-center, pre–post quasi-experimental study	132 patients	Adults	BioFire FilmArray BCID panel
Pettit et al (2019) [6]	Single-center, retrospective, theoretical impact study	149 patients	Adults	BioFire FilmArray (FA) and Nanosphere Verigene (NV)
Martin et al (2024) [7]	Single-center, retrospective cohort study	253 positive blood culture episodes (153 with BCID, 100 with standard techniques)	Adults	BioFire FilmArray BCID panel
Beal et al (2015) [8]	Quasi-experimental	168 episodes of bacteremia screened	Adults	Verigene GP-BC
Buss et al (2018) [9]	Quasi-experimental	52 pre/43 post	Adults	BioFire FilmArray
Nakagawa et al (2018) [10]	Quasi-experimental	44 pre/20 post	Adults	Verigene GP-BC
Bandy et al (2023) [11]	Quasi-experimental	50 pre/54 post	Adults	Verigene GP-BC
Gawrys et al (2020) [12]	Quasi-exp	68 pre/73 post	Adults	Verigene GN-BC
MacVane et al (2016) [13]	Quasi-exp	115/104/145	Adults	BCID
Rivard et al (2017) [14]	Quasi-exp	456/421	Adults	Verigene GN-BC
Banerjee et al (2015) [15]	Randomized controlled trials	207/198/212	Adults	BCID
Tritle et al (2022) [16]	Quasi-exp	94 pre/172 post	Adults	BCID
Box et al (2015) [17]	Quasi-experimental	103/64	Adults	Verigene GP-BC
Tseng et al (2018) [18]	Quasi-exp	103/100	Adults	BCID
Goshorn et al (2023) [19]	Quasi-exp	65/60/57	Adults	ePlex System
Roshdy et al (2015) [20]	Quasi-exp	65 pre/74 post	Adults	Verigene GP-BC
Sango et al (2013) [21]	Quasi-exp	46/28	Adults	Verigene GP-BC
Claeys et al (2020) [22]	Retrospective Quasi-exp	237/308/237	Adults	Verigene GN-BC

### Data Extraction, Management, and Analysis

Two reviewers independently extracted data using a standardized form. Meta-analyses used random-effects models (DerSimonian-Laird) to estimate pooled odds ratios, mean differences, or standardized effect sizes with 95% CIs. Median/IQR values were converted to means/SDs [23]. Heterogeneity was assessed using I^2^ and Cochran's Q test. Prespecified sensitivity analyses included leave-one-out analysis and Hartung-Knapp reanalysis; subgroup analyses and meta-regression explored heterogeneity sources. Prediction intervals were calculated for principal outcomes to estimate the expected range of effects in future similar settings. Trial sequential analysis was performed for TTAT (time to appropriate therapy), LOS, and mortality to assess whether cumulative evidence crossed monitoring boundaries after adjustment for accrued information size. Additional methods are provided in the Supplementary Appendix.

### Risk of Bias Assessment

Risk of bias was assessed using RoB 2 for the randomized trial and ROBINS-I for nonrandomized studies, evaluating 6 domains: confounding, selection, intervention classification, missing data, outcome measurement, and reporting. Meta-regression assessed whether study quality influenced pooled estimates. Detailed assessments are provided in the Supplementary Appendix.

## RESULTS

### Study Selection

Study selection is shown in the PRISMA flow diagram (Figure 1)

### Risk of Bias Within Studies

Of 20 included studies, 6 (30%) had low overall risk of bias, 12 (60%) moderate, and 1 (5%) serious; none were critical (Table 2). The randomized trial had “some concerns.” The most common bias source was confounding (55% at serious risk), reflecting predominant pre–post designs. Meta-regression showed study quality did not significantly predict effect sizes for any outcome (all *P* > .08). Detailed assessments are in Supplementary Results 1. Because most studies were observational or pre–post implementation studies, residual confounding, and secular trends remain important limitations despite sensitivity analyses.

**Table 2.** Risk of Bias Within Studies

Study	Tool	Overall Risk of Bias	NOS-equivalent Score
Banerjee et al (2015) [15]	RoB 2	Some concerns	7
Donnelly et al (2021) [4]	ROBINS-I	Moderate	7
Chiasson et al (2022) [1]	ROBINS-I	Moderate	6
Russo et al (2019) [5]	ROBINS-I	Serious	4
Pettit et al (2019) [6]	ROBINS-I	Moderate	6
Martin et al (2024) [7]	ROBINS-I	Moderate	6
Beal et al (2015) [8]	ROBINS-I	Moderate	6
Buss et al (2018) [9]	ROBINS-I	Low	8
Nakagawa et al (2018) [10]	ROBINS-I	Moderate	6
Bandy et al (2023) [11]	ROBINS-I	Moderate	6
Gawrys et al (2020) [12]	ROBINS-I	Moderate	6
MacVane et al (2016) [13]	ROBINS-I	Moderate	6
Rivard et al (2017) [14]	ROBINS-I	Low	8
Tritle et al (2022) [16]	ROBINS-I	Low	8
Box et al (2015) [17]	ROBINS-I	Moderate	6
Tseng et al (2018) [18]	ROBINS-I	Low	8
Goshorn et al (2023) [19]	ROBINS-I	Low	8
Roshdy et al (2015) [20]	ROBINS-I	Moderate	6
Sango et al (2013) [21]	ROBINS-I	Moderate	6
Claeys et al (2020) [22]	ROBINS-I	Low	8

ROBINS-I was used for nonrandomized comparative studies and RoB 2 was used for the randomized trial by Banerjee et al (2015) [15]. NOS-equivalent scores were derived using a prespecified crosswalk for exploratory sensitivity analyses and should be interpreted as approximate rather than as formal rescoring.

### Results of Syntheses

#### Time-to-Appropriate Therapy

**Figure 2.**
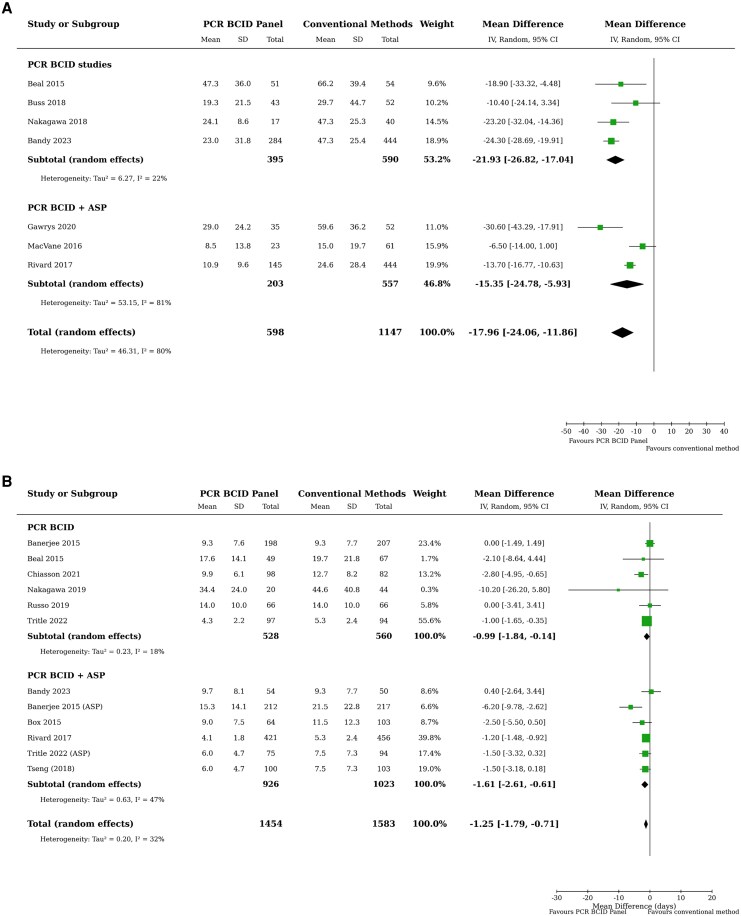
(*A*) Pooled effect size for time-to-appropriate therapy and forest plot; (*B*) pooled effect size of length of stay and forest plot.

Seven studies (n = 1453 patients) assessed time to appropriate therapy. BCID implementation significantly reduced time to appropriate therapy by 17.28 hours (MD −17.28, 95% CI −24.00 to −10.56; Figure 2*A*). Heterogeneity was substantial (I^2^ = 81.8%). The finding remained robust in leave-one-out analyses and Hartung-Knapp reanalysis (MD −17.28 hours, 95% CI −24.81 to −9.75; *P* = .0014).

#### Sensitivity and Robustness Analyses

Leave-one-out analyses confirmed that the primary findings were not driven by any individual study. For time to appropriate therapy, exclusion of MacVane et al (2016) produced the greatest heterogeneity reduction (I^2^ 81.8% to 59.8%). For LOS, exclusion of Banerjee et al (2015) (ASP) reduced I^2^ from 32.3% to 0.0%. Hartung-Knapp reanalysis confirmed significant reductions in time to appropriate therapy (*P* = .0014) and LOS (*P* = .0021), while stewardship outcomes were attenuated (*P* = .07) and economic outcomes remained nonsignificant (*P* = .15). Prediction intervals remained favorable for TTAT (−33.96 to −0.60 hours) and LOS (−2.29 to −0.22 days), whereas the mortality interval crossed the null, indicating uncertainty in expected mortality effects across future similar settings. Trial sequential analysis showed that cumulative evidence for TTAT and LOS crossed O’Brien-Fleming monitoring boundaries, whereas mortality crossed neither efficacy nor harm boundaries. Complete results are in Supplementary Results 2 and Supplementary Table 1.

#### Subgroup Analyses

Prespecified subgroup analyses were performed to explore sources of heterogeneity and identify contexts in which BCID panels demonstrated greatest benefit.

Pathogen specificity significantly modified time to appropriate therapy effects (χ^2^ = 25.73, *P* < .0001). Gram-positive-specific studies showed the largest reduction (−22.55 hours; I^2^ = 0%), likely reflecting rapid methicillin- and vancomycin-resistance detection. Gram-negative studies showed variable effects (−21.15 hours; I^2^ = 82.5%). BCID platform type also modified TTAT effects (χ^2^ = 9.03, *P* = .003): Verigene-based studies showed larger reductions (−21.41 hours) than BioFire (−7.39 hours). Platform type did not significantly modify LOS, mortality, stewardship, or economic outcomes. Implementation model (BCID alone vs BCID + ASP) did not show significant effect modification for time to appropriate therapy (*P* = .92) or LOS (*P* = .35). Complete subgroup results, including study counts by platform and outcome, are in Supplementary Results 3 and Supplementary Table 1.

#### Clinical Outcomes

##### Length of Stay

Twelve studies (n = 3037) reported LOS. BCID implementation reduced LOS by 1.25 days (MD −1.25, 95% CI −1.79 to −.71; I^2^ = 32.3%; Figure 2*B*).

#### Mortality

**Figure 3.**
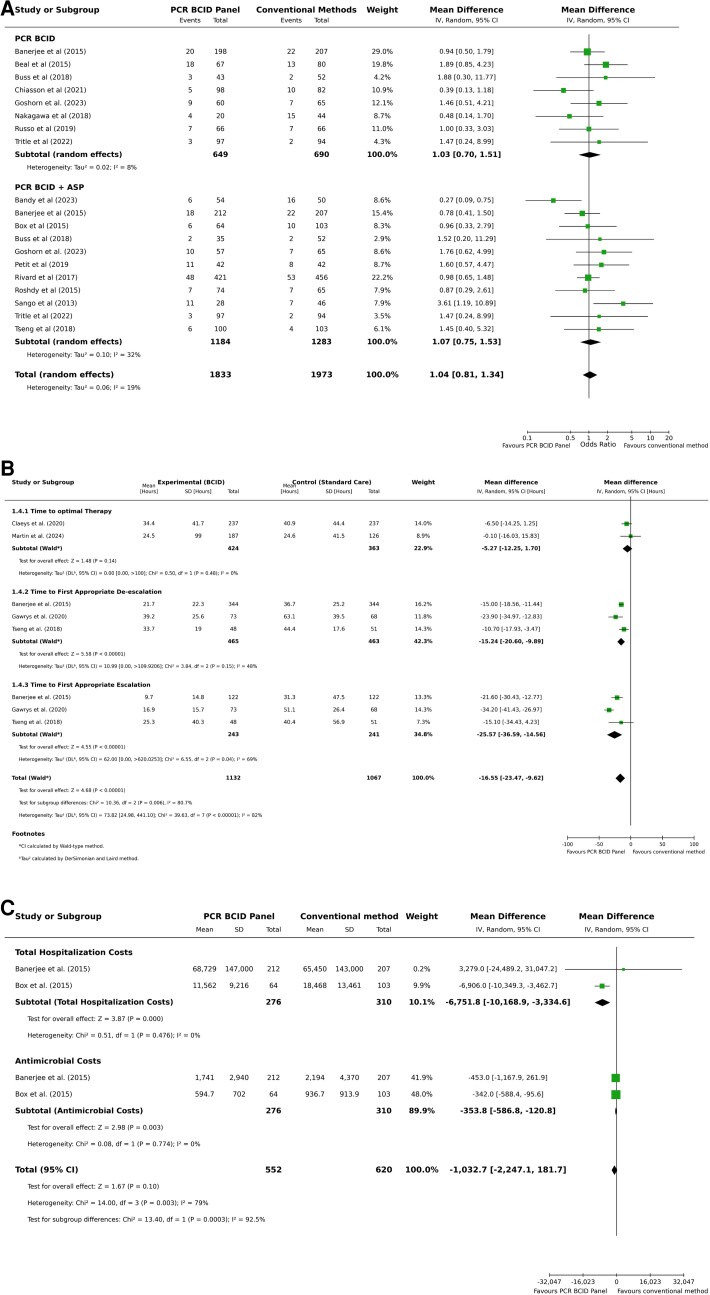
(*A*) Pooled odds ratio for mortality and forest plot; (*B*) pooled results for key antimicrobial stewardship metrics and forest plot; (*C*) pooled economic outcomes of multiplex PCR–guided care versus standard care.

Nineteen studies (n = 3806) reported mortality. BCID panels were not associated with significant mortality reduction (OR 1.04, 95% CI .81–1.34; *P* = .74; I^2^ = 19%; Figure 3*A*). Subgroup analyses did not identify effect modification.

#### Antimicrobial Stewardship

Eight studies assessed stewardship metrics, defined as study-reported time to de-escalation, time to escalation, or time to antimicrobial optimization, and analyzed separately from TTAT (Figure 3*B*). The pooled reduction was 16.55 hours (95% CI −23.47 to −9.62; *P* < .00001; I^2^ = 82%). Subgroup analysis revealed significant differences by outcome type (*P* = .006): time to de-escalation was reduced by 15.24 hours (*P* < .00001), and time to escalation by 25.57 hours (*P* < .00001), while time to optimization showed nonsignificant reduction (−5.27 hours, *P* = .14). However, in Hartung-Knapp reanalysis, the pooled effect was attenuated (Hedges' g 0.46, 95% CI −.07 to 1.00; *P* = .07). Details are in Supplementary Results 6.

#### Economic Outcomes

Four studies provided observed economic data. The pooled effect was not significant (Hedges' g 0.24, 95% CI −.18 to .67; *P* = .26; I^2^ = 75.8%; Figure 3*C*), remaining nonsignificant in Hartung-Knapp reanalysis. Details are in Supplementary Results 7. Using GRADE, certainty was moderate for TTAT, LOS, and mortality, low for stewardship outcomes, and very low for economic outcomes (Table A1).

Exploratory analyses suggested that implementation intensity did not significantly contribute to between-study variability for TTAT and LOS, whereas no single meta-regression covariate independently explained heterogeneity in the main clinical outcomes.

#### Publication Bias

Funnel plots and formal tests (Egger's and Begg's) did not provide strong evidence of publication bias for time to appropriate therapy (*P* = .700), LOS (*P* = .334), or mortality (*P* = .536). Trim-and-fill analysis did not alter the mortality estimate. Funnel plots are in Supplementary Figures 1-3.

## DISCUSSION

This meta-analysis found that multiplex PCR BCID panels were associated with reduced time to appropriate therapy (−17.28 hours) and LOS (−1.25 days) in US hospitals. These findings were robust across sensitivity analyses, prediction intervals, and trial sequential analysis. In contrast, no mortality benefit was observed, stewardship estimates were heterogeneous and less robust, and economic effects were nonsignificant (Hedges' g 0.24, 95% CI −.18 to .67). Certainty of evidence was moderate for TTAT and LOS, supporting clinical relevance of these process outcomes, but low or very low for stewardship and economic outcomes, requiring cautious interpretation.

The 17.28-hour reduction in time to appropriate therapy is clinically meaningful. Van Heuverswyn et al (2023) identified a critical 12-hour threshold beyond which inappropriate therapy significantly increases 30-day mortality (adjusted OR 1.17, 95% CI 1.01–1.37), with risk escalating at 24, 48, and 72 hours [24]. Lee et al (2019) reported 0.3% increased mortality per hour of delay (adjusted OR 1.003), rising to 0.4% in critically ill patients [25]. Our observed reduction may move patients from high-risk delayed-therapy categories into lower-risk early therapy windows, potentially translating to approximately 5% mortality reduction—an effect that may be undetectable in underpowered individual studies.

Subgroup analyses revealed largest time-to-appropriate-therapy reductions in Gram-positive bacteremia (−22.55 hours), likely reflecting rapid methicillin-resistance detection enabling prompt de-escalation from empiric vancomycin in MSSA or escalation in VRE infections. Gram-negative effects were more variable (−21.15 hours, I^2^ = 82.5%), possibly because empiric broad-spectrum coverage often provides adequate initial therapy and because included studies varied in Gram-negative resistance-marker coverage. Several included assays, including Verigene GN-BC and ePlex BCID-GN, include clinically relevant Gram-negative resistance markers, including ESBL-associated and carbapenemase genes. Newer or expanded panels may provide greater benefit in settings with increasing antimicrobial resistance, but the included evidence was insufficient for a definitive marker-level or panel-generation subgroup analysis. BCID panels are likely to be most valuable in healthcare settings where rapid resistance-marker detection can change early therapy.

As Lodise et al (2020) demonstrated, a delay in providing appropriate treatment increases hospitalization LOS from 6 to 11 days, decreases discharge-to-home rates from 58% to 43%, and increases costs [26]. The 17.28-hour reduction we have observed (approximately 0.7 day) will allow for earlier initiation of therapy, thereby contributing to the 1.25-day reduction in LOS.

The lack of mortality benefits associated with this intervention is consistent with the ASM 2025 guidelines, which assessed the quality of available mortality data as poor due to the limited number of RCTs. Timbrook et al (2017) demonstrated that mortality was significantly reduced (OR = 0.66) in studies with implemented stewardship programs, suggesting that the ability to obtain a diagnosis quickly does not guarantee a clinical response [27, 28]. Peri et al (2024) demonstrated that use of RDT + ASP resulted in a lower mortality rate than did blood cultures alone (OR = 0.72, 95% CI .59–.87) [29]. The lack of a mortality benefit in our study may be attributed to: (1) the variable intensity of the ASP programs used across the studies included in our analysis; (2) the low baseline mortality rates in our study population resulting in an inability to detect a difference in mortality between groups; or (3) the fact that many of the control groups in the included studies achieved the appropriate empiric therapy before the 12 hours following admission to the hospital, thereby reducing the opportunity for additional benefit [29].

In addition, the potential for a mortality benefit may be pathogen-specific. Ohnuma et al (2023) demonstrated that appropriate empiric therapy can reduce mortality in Pseudomonas aeruginosa (OR = 0.51) and *Klebsiella pneumoniae* (OR = 0.67) but not in *Escherichia coli* (OR = 0.93). Thus, the benefit of using BCID panels may be greatest when empiric coverage is inadequate [30].

The gap between how quickly a diagnosis can be made through rapid diagnostic testing (1–8 hours) and the real-world changes to practice and process required to implement it reflects the many challenges to implementing rapid diagnostic testing beyond the technology itself. This response gap may explain why BCID implementation consistently improved process measures such as TTAT and LOS, while mortality remained unchanged; diagnostic acceleration alone may be insufficient unless results are paired with active communication, stewardship interpretation, and timely clinician action. For example, Stewart et al (2025) indicated that a clinician's “mental lines” or “thinking patterns” are frequently overriding the results of a test, where clinicians have stated, “I will take a conservative approach and provide broad-spectrum antibiotics regardless of whether the organism tested was resistant or not.” [31]. Furthermore, Burrowes et al (2021) demonstrated that clinicians are more likely to rely on their own clinical judgment regarding appropriate treatment than on the results of a rapid diagnostic test [32]. Additionally, the IDSA/SHEA Guidelines (2016) noted that simply reporting laboratory data (ie, test results) does not change antibiotic selection practices; therefore, some form of active stewardship intervention is required [32, 33]. These factors help explain why the LOS reductions seen in the BCID + ASP group (−1.61 days) were numerically greater than those seen in the BCID group alone (−0.99 days); however, this difference was not statistically significant.

BCID panels enable stewardship teams to intervene in the early stages of disease, when the likelihood of therapeutic success is greatest. In addition to the ability to initiate prompt narrow-spectrum therapy for susceptible organisms, the significant time reductions in de-escalation (−15.24 hours) and escalation (−25.57 hours) also support the dual function of BCID panels.

The modest nonsignificant pooled economic impact (Hedges' g = 0.24) should be viewed with caution because only 4 studies contributed to this analysis, costing methods varied, and most economic evaluations were embedded within observational pre–post designs. Pliakos et al (2018) demonstrated that MALDI-TOF with ASP is expected to have an 80% chance of being cost-effective, compared with 41% for MALDI-TOF alone, underscoring the need for a coordinated stewardship program to realize cost savings [34]. The costs of LOS and daily hospitalizations were the primary factors influencing cost-effectiveness in the study conducted by Mponponsuo et al (2022) [35]. Therefore, potential savings inferred from shorter LOS should be considered exploratory and hypothesis-generating rather than definitive evidence of economic benefit. However, MacVane and Nolte (2016) indicated that adding BCID to a robust ASP with low resistance rates would incur no additional cost, suggesting that cost savings will depend on the setting [13].

This meta-analysis has several limitations. First, the majority of the evidence reported here was based on observational studies, which increase the risk of confounding. Second, the implementation of these programs' components varies significantly, from minimal stewardship support to a complete ASP bundle. In addition, platform-specific Gram-negative resistance-marker detection, ESBL/CRE prevalence, and marker-positive subgroup outcomes were not consistently reported, limiting marker-level analyses. Third, restriction to English-language US-based studies, most from large academic centers, may limit generalizability to non-US healthcare systems and smaller community hospitals. Fourth, substantial heterogeneity persisted for time to appropriate therapy and economic outcomes. Finally, economic analysis was limited to 4 studies with directly observed costs, and the pooled effect was nonsignificant with substantial heterogeneity. Future studies should evaluate patient-centered and stewardship-specific outcomes not consistently reported in the current literature, including time to oral switch when appropriate, antibiotic-related adverse drug events, *Clostridioides difficile* infection, avoidance of unnecessary long-term intravenous access, and PICC placement.

## CONCLUSION

Multiplex PCR BCID panels were associated with shorter time to appropriate therapy by 17.28 hours and LOS by 1.25 days. Although no mortality benefit was demonstrated, these process improvements may have greatest impact within the critical 12-hour window. BCID panels should be implemented as part of comprehensive antimicrobial stewardship programs that translate rapid diagnostic information into timely therapeutic action.

## Supplementary Material

ofag370_Supplementary_Data

## Supplementary Data

Supplementary materials are available at *Open Forum Infectious Diseases* online. Consisting of data provided by the authors to benefit the reader, the posted materials are not copyedited and are the sole responsibility of the authors, so questions or comments should be addressed to the corresponding author.

## APPENDIX

### Table A1. Grading the Certainty of Evidence

**Table:**

Outcome	Participants (Studies)	Risk of Bias	Inconsistency	Indirectness	Imprecision	Publication Bias	Overall Certainty	Effect (95% CI)
Time to appropriate therapy	1453 (7 nonrandomized)	Serious^a^	Very serious^b^	Not serious	Not serious	Not detected	⨁⨁⨁◯ Moderate	MD 17.28 h fewer (24.00 fewer to 10.56 fewer)
Length of stay	3037 (12 nonrandomized)	Serious^c^	Not serious^d^	Not serious	Not serious	Not detected	⨁⨁⨁◯ Moderate	MD 1.25 d fewer (1.79 fewer to 0.71 fewer)
Mortality	3806 (19 studies, predominantly nonrandomized)	Serious^e^	Not serious	Not serious	Not serious	Not detected	⨁⨁⨁◯ Moderate	OR 1.04 (0.81 to 1.34) 44 more per 1000 (21 fewer to 58 more)
Antimicrobial stewardship	2199 (8 nonrandomized)	Serious^f^	Serious^g^	Not serious	Serious^h^	Not detected	⨁⨁◯◯ Low	MD 16.55 h fewer (23.47 fewer to 9.62 fewer)
Economic outcomes	1172 (4 nonrandomized)	Serious^i^	Serious^j^	Not serious	Serious^k^	Unable to assess^l^	⨁◯◯◯ Very low	Hedges' g 0.24 (−0.18 to 0.67) HK: 0.24 (−0.16 to 0.64)

Implementation of a multiplex PCR blood culture identification (BCID) panel integrated into clinical care pathways compared with conventional microbiological methods in hospitalized adults with bloodstream infection.

^a^(Risk of bias) All included studies were nonrandomized (pre–post or cohort) and were therefore vulnerable to confounding, particularly by time and secular trends. This justified rating down for risk of bias.

^b^(Inconsistency) Heterogeneity for time to appropriate therapy remained considerable, with substantial between-study variability in effect magnitude despite a consistent direction of benefit. This justified rating down 2 levels.

^c^(Length of stay risk of bias) Length of stay is highly susceptible to confounding by patient mix, comorbidities, discharge practices, and secular trends unrelated to the intervention. The nonrandomized design of the included studies justified rating down.

^d^(Length of stay inconsistency) Heterogeneity for length of stay was low in the updated analysis, with a consistent direction of effect favoring BCID implementation across studies. Therefore, inconsistency was not considered serious.

^e^(Mortality risk of bias) The evidence base for mortality was predominantly observational, and mortality is susceptible to confounding by illness severity, comorbidities, and cointerventions.

^f^(Antimicrobial stewardship risk of bias) Stewardship process metrics are vulnerable to confounding by concurrent quality-improvement initiatives and changes in prescribing culture over time.

^g^(Antimicrobial stewardship inconsistency) There was considerable heterogeneity across stewardship outcomes, and the observed effects varied across different stewardship actions.

^h^(Antimicrobial stewardship imprecision) The apparent benefit was attenuated and no longer clearly significant in Hartung-Knapp reanalysis, reducing confidence in precision of the estimate.

^i^(Economic outcomes risk of bias) Economic outcomes are vulnerable to confounding by case-mix severity, payer mix, hospital accounting practices, and concurrent cost-containment initiatives.

^j^(Economic outcomes inconsistency) Substantial heterogeneity remained after sensitivity analyses and was not fully explained by any single remaining study.

^k^(Economic outcomes imprecision) The pooled estimate crossed the line of no effect and remained nonsignificant in Hartung-Knapp reanalysis.

^l^(Economic outcomes publication bias) Formal assessment was limited by the small number of included studies, so small-study effects could not be excluded reliably.
