# Supplementary material for: Impact of Multiplex PCR Blood-Culture Identification Panels on Clinical Outcomes, Antimicrobial Stewardship, and Economic Impact in US Hospitals: A Systematic Review and Meta-Analysis
Source: Open Forum Infect Dis. 2026 Jun 25;13(7):ofag370. doi: 10.1093/ofid/ofag370 (PMC13332404; doi:10.1093/ofid/ofag370)
Supplement: ofag370_Supplementary_Data [file ofag370_supplementary_data.zip › Appendix OFID_revised May 27-2026.docx]

**Supplementary Appendix**

**Impact of Multiplex PCR Blood-Culture Identification Panels on Clinical Outcomes, Antimicrobial Stewardship and Burden of Cost in U.S. Hospitals: A Systematic Review and Meta-Analysis**

Muqtadir J, Bhatti S, Batool I, Lesho EP

**Table of Contents**

1. Search Strategy (Methods S1)

2. Data Extraction Fields (Methods S2)

3. Additional Statistical Methods (Methods S3)

4. Risk of Bias Assessment (Results S1)

5. Sensitivity and Exploratory Analyses (Results S2)

6. Subgroup and Meta-Regression Analyses (Results S3)

7. Additional Time-to-Appropriate Therapy Findings (Results S4)

8. Additional Mortality Findings (Results S5)

9. Additional Antimicrobial Stewardship Results (Results S6)

10. Additional Economic Results (Results S7)

11. Publication Bias Assessment (Results S8)

12. Temporal Stability Analysis (Results S9)

13. Supplementary Figures (Figures S1–S7)

14. Extended Discussion (Discussion S1–S6)

**SUPPLEMENTARY METHODS**

**1. Methods S1. Full Search Strategy**

Six electronic databases were searched systematically to identify all studies assessing the use of rapid diagnostic panels for diagnosing bloodstream infections. All searches were conducted by November 15, 2025. MeSH headings and keywords were used with Boolean operators to create the search strategy. No date restrictions were applied; the search was limited to studies published in English.

**PubMed:**

(("multiplex PCR"[Title/Abstract] OR "rapid diagnostic"[Title/Abstract] OR "FilmArray"[Title/Abstract] OR "Verigene"[Title/Abstract] OR "ePlex"[Title/Abstract] OR "BCID"[Title/Abstract] OR "blood culture identification"[Title/Abstract]) AND ("bloodstream infection"[Title/Abstract] OR "bacteremia"[Title/Abstract] OR "sepsis"[Title/Abstract] OR "fungemia"[Title/Abstract] OR "Blood Culture"[MeSH Terms]) AND ("antimicrobial stewardship"[Title/Abstract] OR "time to therapy"[Title/Abstract] OR "time to appropriate therapy"[Title/Abstract] OR "length of stay"[Title/Abstract] OR "mortality"[Title/Abstract] OR "cost"[Title/Abstract] OR "Antimicrobial Stewardship"[MeSH Terms]))

**Embase:**

('multiplex polymerase chain reaction'/exp OR 'rapid diagnostic test'/exp OR 'FilmArray':ti,ab OR 'Verigene':ti,ab OR 'ePlex':ti,ab OR 'BCID':ti,ab) AND ('bacteremia'/exp OR 'sepsis'/exp OR 'bloodstream infection':ti,ab OR 'fungemia':ti,ab) AND ('antimicrobial stewardship'/exp OR 'time to therapy':ti,ab OR 'length of stay':ti,ab OR 'mortality':ti,ab OR 'cost effectiveness':ti,ab)

**Scopus:**

TITLE-ABS-KEY(("multiplex PCR" OR "rapid diagnostic" OR "FilmArray" OR "Verigene" OR "ePlex" OR "BCID") AND ("bloodstream infection" OR "bacteremia" OR "sepsis" OR "fungemia") AND ("antimicrobial stewardship" OR "time to therapy" OR "length of stay" OR "mortality" OR "cost"))

**CINAHL:**

(MH "Polymerase Chain Reaction" OR "multiplex PCR" OR "rapid diagnostic" OR "FilmArray" OR "Verigene" OR "ePlex" OR "BCID") AND (MH "Bacteremia" OR MH "Sepsis" OR "bloodstream infection" OR "fungemia") AND (MH "Antimicrobial Stewardship" OR "time to therapy" OR "length of stay" OR "mortality" OR "cost")

**Web of Science:**

TS=(("multiplex PCR" OR "rapid diagnostic" OR "FilmArray" OR "Verigene" OR "ePlex" OR "BCID") AND ("bloodstream infection" OR "bacteremia" OR "sepsis" OR "fungemia") AND ("antimicrobial stewardship" OR "time to therapy" OR "length of stay" OR "mortality" OR "cost"))

**Cochrane Library:**

("multiplex PCR" OR "rapid diagnostic" OR "FilmArray" OR "Verigene" OR "ePlex" OR "BCID") AND ("bloodstream infection" OR "bacteremia" OR "sepsis") AND ("antimicrobial stewardship" OR "time to therapy" OR "length of stay" OR "mortality" OR "cost")

**2. Methods S2. Data Extraction Fields**

Two reviewers independently extracted data using a standardized, pre-piloted form; discrepancies were resolved through discussion. Extracted data included: study identifiers (first author, year, journal, country); study characteristics (design, period, setting, single vs. multi-center); population characteristics (sample size, demographics, inclusion/exclusion criteria, pathogen distribution, severity indicators); intervention details (BCID platform type, implementation model, ASP intensity score, laboratory workflow, notification method); comparator description (conventional methods, baseline ASP presence); outcome data (time to appropriate therapy, length of stay, mortality, stewardship metrics, economic outcomes); and effect measures (point estimates with variance, adjusted vs. unadjusted estimates, covariates).

1. **3. Methods S3. Additional Statistical Methods**

   **Random-Effects Meta-Analysis:**

   Random effects meta-analysis was conducted using the DerSimonian-Laird weighting method to estimate the pooled odds ratios, weighted mean differences, or standardized effect sizes (Hedges'g) with 95% confidence intervals for the specified outcomes. The random-effects model was chosen a priori because it was expected that both clinical and methodological heterogeneity would be present among the included studies.

   **Median-to-Mean Conversion:**

   Studies that reported results as medians and Interquartile Range (IQR) instead of means and Standard Deviations (SD) had their means and SDs estimated using the method described by Wan et al. (2014). A sensitivity analysis that excluded studies with converted statistics was performed to assess the robustness of the estimates.

   **Heterogeneity Assessment:**

   The degree of between-study heterogeneity was assessed using the I² statistic and the Cochran's Q-test. For I² values of 25%, 50%, and 75%, these correspond to low, moderate, and high levels of heterogeneity, respectively. However, due to the Q-test's low power, P-values > 0.1 were used to indicate significant heterogeneity.

   **Sensitivity Analyses:**

   - **Leave-one-out analysis:** Each study was sequentially omitted to determine how each study influenced the pooled estimate and the level of heterogeneity.

   - **Hartung-Knapp-Sidik-Jonkman adjustment:** This was applied to provide more conservative confidence intervals that take into account the uncertainty associated with estimating the variance of the heterogeneity, especially when the number of studies is small for a particular outcome.

   - **Fixed-effect vs. random-effects comparison:** The sensitivity of the conclusion to the choice of model was assessed.

   - **Median-to-mean conversion exclusion:** Exclusion of studies that have been clearly identified to have median to mean conversions.

   **Subgroup Analyses:**

   Prespecified subgroup analyses were performed by:

1. Implementation Model (BCID-alone vs. BCID+ASP)

2. ASP Intensity (Low, Moderate, High, based on pre-specified scores)

3. Pathogen Specificity (Gram Positive, Gram-negative, Unspecified)

4. Type of BCID Platform Used (BioFire, Verigene, ePlex)

5. Design of the Study (Prospective vs. Retrospective)

Chi-square tests were used to evaluate differences in estimates across subgroups. Additionally, the proportion of heterogeneity accounted for by the subgroup variables was also evaluated.

**Meta-Regression:**

Univariable random-effects meta-regressions were conducted using Restricted Maximum Likelihood (REML) on prespecified study-level covariates that potentially explain variability in effect sizes across studies (i.e., between-study heterogeneity). These potential explanatory variables included: a continuous variable – the ASP intensity score; categorical variables – Implementation Model, Pathogen Specificity, and Categorical BCID Platform Type. A measure of the proportion of the variability explained by each predictor was assessed via R². Multivariable meta-regression was explored as an additional analysis (and prespecified) to determine the joint effects of the ASP Intensity Score and Implementation Model on Length of Stay, regardless of whether individual predictors achieved statistical significance at the conventional threshold.

**Influence Diagnostics:**

Explicit influence diagnostics included:

1. Externally studentized residuals (outlier detection)

2. Cook's distances (overall influence)

3. Leverage values (influence on model fit)

4. Baujat plots (contribution to heterogeneity vs. influence on pooled effect)

**Risk of Bias Sensitivity Analyses:**

1. Meta-regression using continuous NOS-equivalent quality scores

2. Meta-regression using categorical overall risk-of-bias ratings

3. Domain-specific moderator analyses (confounding, selection, outcome measurement)

4. Quality-effects modeling incorporating both study precision and quality weighting

**Publication Bias Assessment:**

The presence of publication bias was evaluated via visual inspection of funnel plots; the use of Eggers' regression test and Begg and Mazumdar's rank correlation test (where feasible) for evaluation of funnel plot asymmetry. Due to the limited power of the statistical tests for assessing funnel plot asymmetry when there are less than ten studies in the outcome being evaluated, the results from these analyses were viewed with caution and considered exploratory. As there were nineteen studies evaluating mortality, Duval and Tweedie's trim-and-fill analysis was also evaluated as an alternative method for sensitivity analysis. Contour-enhanced funnel plots that included significance contours were utilized for evaluation of the extent to which any apparent asymmetry in the funnel plots could be explained by publication bias versus other study-level small-study effects.

**Cumulative Meta-Analysis:**

Cumulative chronological meta-analyses were conducted by sequentially adding each study to the pool in the order of publication to evaluate the temporal stability of the estimated effect size.

**Prediction Intervals:**
Prediction intervals were calculated for the principal random-effects meta-analyses to estimate the range of effects expected in a future similar study. Prediction intervals were generated for time to appropriate therapy, length of stay, and mortality.

**Trial Sequential Analysis:**
Trial sequential analyses were performed for time to appropriate therapy, length of stay, and mortality. DARIS was calculated using prespecified clinically important differences of 6 hours for time to appropriate therapy and 1 day for length of stay, and a 20% relative risk reduction for mortality, with two-sided α=0.05 and 80% power. Required information sizes were adjusted for between-study heterogeneity, and cumulative Z-curves were assessed against O’Brien-Fleming monitoring boundaries.

**SUPPLEMENTARY RESULTS**

**4. Results S1. Risk of Bias Assessment**

Risk of bias was assessed using ROBINS-I for non-randomized studies and RoB 2 for the randomized trial (Banerjee et al. 2015). Across the 20 included studies: 30% (6 studies) had low overall risk of bias, 60% (12 studies) had moderate risk, 5% (1 study) had serious risk, and no study was rated as critical risk. The single randomized study was judged as having some concerns. The most common source of bias was confounding (55% of studies at serious risk), reflecting the predominance of pre-post quasi-experimental designs.

**Risk of Bias Sensitivity Analyses:**

Meta-regression using NOS-equivalent scores as a continuous quality predictor did not identify significant associations between study quality and effect size for any outcome (all P > 0.08). Similarly, meta-regression using categorical risk-of-bias ratings and domain-specific moderator analyses did not identify significant effect modification. In exploratory quality-effects models, pooled effects for time to appropriate therapy (Hedges' g 0.70, 95% CI 0.57–0.83), length of stay (Hedges' g 0.33, 95% CI 0.24–0.41), and stewardship outcomes (Hedges' g 0.45, 95% CI 0.36–0.55) remained statistically significant, while mortality remained non-significant (log OR 0.04, 95% CI −0.20 to 0.28).
Overall, domain-specific sensitivity analyses did not identify a single ROBINS-I domain that significantly explained heterogeneity, suggesting that between-study variability was multifactorial rather than driven by any single dominant bias domain.

**5. Results S2. Sensitivity and Exploratory Analyses**

Multiple sensitivity analyses were performed to assess robustness of pooled estimates. Results are summarized in Supplementary Table S1.

**Leave-One-Out Sensitivity Analyses:**

The combined effect on time to appropriate therapy (TTAT) was significant even after sequentially removing studies. The largest decrease in variance occurred when MacVane et al. (2016) were removed from the analysis, with a decrease in the measure of heterogeneity from 81.8 percent to 59.8 percent. When combining the data related to length of stay (LOS), the overall effect size remained significant, although excluding ASP from Banerjee et al. (2015) resulted in a drop in the measure of heterogeneity from 32.3 percent to 0.0 percent. The results indicated that none of the studies alone could explain all the variation in the data related to economic outcomes. Excluding Box et al. (2015) resulted in a drop in the measure of heterogeneity from 75.8 percent to 61.8 percent, however the overall effect size remained non-significant. Excluding Gawrys et al. (2020) resulted in a drop in the measure of heterogeneity from 93.2 percent to 90.2 percent.

**Hartung-Knapp Reanalysis:**

TTAT (MD −17.28 h, 95% CI −24.81 to −9.75; P = 0.0014) and LOS (MD −1.25 days, 95% CI −1.95 to −0.56; P = 0.0021) remained significant. Economic outcomes (Hedges' g 0.24, 95% CI −0.16 to 0.64; P = 0.15) remained non-significant, and stewardship outcomes (Hedges' g 0.46, 95% CI −0.07 to 1.00; P = 0.07) were attenuated.

**Fixed-Effect vs. Random-Effects Comparison:**

Both models yielded similar conclusions for TTAT and LOS, supporting stability of findings despite model choice.

**Median-to-Mean Conversion Sensitivity Analysis:**

Excluding studies with median-to-mean conversion did not materially alter pooled estimates for TTAT (MD −17.10 h, 95% CI −24.53 to −9.66) or length of stay (MD −1.26 days, 95% CI −1.83 to −0.69).

**Influence Diagnostics:**

The MacVane et al. (2016) study accounted for the majority of the variation in TTAT, while the Gawrys et al. (2020) study had the largest effect on the overall effect size estimate. For LOS, Banerjee et al. (2015) (ASP) had the largest effect on LOS estimate variability. In terms of economic outcomes, no individual study was clearly identified as an outlier; however, the Baujat plots confirmed that the majority of the variance in each outcome was attributable to only a few of the included studies and was not uniformly distributed across all studies.
Overall, the benefits for time to appropriate therapy and length of stay were robust, whereas economic and stewardship estimates were more sensitive to influential studies and heterogeneity.

**6. Results S3. Subgroup and Meta-Regression Analyses**

**Subgroup Analysis by ASP Intensity:**

Exploratory subgroup analyses were performed by ASP intensity using three prespecified score bands (low, moderate, and high).

***Time to appropriate therapy:***

Subgroup differences were not statistically significant (χ² = 0.39, df = 2, P = 0.82). Greater reductions in time to appropriate therapy were observed across all ASP-intensity strata, although the magnitude varied:

- Low intensity (k = 2): **−**15.48 hours (95% CI −25.23 to −5.73; I² = 0%)
- Moderate intensity (k = 1): −18.70 hours (95% CI −25.35 to −12.05; I² = 0%)
- High intensity (k = 4): −17.93 hours (95% CI −28.47 to −7.39; I² = 90.6%)

***Length of stay:***

Subgroup differences were not statistically significant (χ² = 2.32, df = 2, P = 0.31), indicating no significant effect modification by ASP intensity for length of stay:

- Low intensity (k = 2): −0.10 days (95% CI −2.06 to 1.86; I² = 0%)
- Moderate intensity (k = 4): −1.37 days (95% CI −2.08 to −0.66; I² = 27.4%)
- High intensity (k = 6): −1.61 days (95% CI −2.52 to −0.70; I² = 47%)

**Subgroup Analysis by Pathogen Specificity:**

***Time to appropriate therapy:***

The effect on time to appropriate therapy differed significantly across pathogen categories (χ² = 25.73, df = 2; P < 0.0001), and pathogen specificity explained 68.5% of the observed heterogeneity:

- Broad or unspecified populations (k = 2): −6.86 hours (95% CI −10.19 to −3.53; I² = 0%)

- Gram-negative-specific studies (k = 2): −21.15 hours (95% CI −39.43 to −2.87; I² = 82.5%)

- Gram-positive-specific studies (k = 3): −22.55 hours (95% CI −26.45 to −18.65; I² = 0%)

***Length of stay:***

Pathogen specificity did not materially modify the effect on length of stay. Subgroup differences were not significant (χ² = 0.07, df = 2; P = 0.97), and pathogen grouping explained 0.0% of heterogeneity.

- Broad or unspecified populations (k = 6): −1.57 days (95% CI −2.85 to −0.29; I² = 60.6%)

- Gram-negative-specific studies (k = 1): −1.20 days (95% CI −1.48 to −0.92)

- Gram-positive-specific studies (k = 5): −0.97 days (95% CI −1.37 to −0.57; I² = 0%)

***Mortality:***

Pathogen specificity did not significantly influence the mortality effect. The subgroup difference was not significant (χ² = 0.55, df = 2; P = 0.758), and pathogen grouping explained only 3.0% of between-study heterogeneity.

**Subgroup Analysis by BCID Platform Type:**

***Time to appropriate therapy:***

Platform type significantly modified the pooled effect (χ² = 9.03, df = 1; P = 0.003), and BCID platform type explained 30.2% of the observed heterogeneity:

- BioFire-based studies (k = 2): **−**7.39 hours (95% CI −11.82 to −2.96; I² = 0%)

- Verigene-based studies (k = 5): −21.41 hours (95% CI −31.80 to −11.02; I² = 80.6%)

***Length of stay, mortality, economic outcomes, and stewardship outcomes:***
BCID platform type did not significantly modify the pooled effects for these outcomes (all P > 0.05). Platform subgroup findings were interpreted cautiously because resistance-marker menus differ across assays and panel generations. Verigene GN-BC and ePlex BCID-GN include clinically relevant Gram-negative resistance markers, including ESBL-associated and carbapenemase genes; however, included studies did not consistently report outcomes stratified by marker detection, ESBL/CRE phenotype, local resistance prevalence, or panel generation. Therefore, platform subgroup analyses should not be interpreted as definitive evidence of superiority of one platform over another.

**Subgroup Analysis by Study Design:**

For mortality, the pooled effect was similar between prospective studies (k = 18; pooled effect 1.23, 95% CI 0.88 to 1.57; I² = 5.1%) and retrospective studies (k = 1; pooled effect 1.60, 95% CI 0.18 to 3.02), with no significant subgroup difference (χ² = 0.26, df = 1; P = 0.613). Study design explained only 1.4% of between-study heterogeneity.

**Meta-Regression Analyses:**

Univariable random-effects meta-regression analyses were performed to explore whether study-level characteristics explained between-study heterogeneity across the major outcomes.

***Time to appropriate therapy:***

ASP intensity score: β = −0.99 (95% CI −8.94 to 6.96; P = 0.807); R² = 0.0%

Implementation model: β = 5.85 (95% CI −4.78 to 16.47; P = 0.281); R² = 32.6%

***Length of stay:***

ASP intensity score: β = −0.54 (95% CI −1.43 to 0.35; P = 0.233); R² = 0.0%

Implementation model: β = −0.55 (95% CI −1.92 to 0.82; P = 0.432); R² = 0.0%

***Mortality:***

- Implementation model: β = 0.02 (95% CI −0.49 to 0.52; P = 0.948); R² = 24.3%

***Economic outcomes:***

- Cost category: β = 0.01 (95% CI −0.49 to 0.51; P = 0.970); omnibus test P = 0.980

***Stewardship outcomes:***

Stewardship subtype significantly modified the pooled effect. Compared with optimal therapy:

- De-escalation: β = 0.57 (95% CI 0.05 to 1.08; P = 0.032)

- Escalation: β = 0.74 (95% CI 0.22 to 1.27; P = 0.006)

The omnibus moderator test was significant (P = 0.017), and stewardship subtype explained 54.3% of between-study heterogeneity (R² = 54.3%).
 ***Multivariable meta-regression:***

A multivariable meta-regression was performed which included ASP intensity score and implementation model simultaneously. In inverse-variance weighted analysis, neither ASP intensity (β = −1.19, 95% CI −3.52 to 1.15; P = 0.320) nor implementation model (β = 0.97, 95% CI −2.36 to 4.30; P = 0.569) was independently associated with effect size. The weighted model explained 0.0% of the variability in effect size, although the omnibus test did not reach statistical significance (P = 0.452).

Because no significant univariable predictors were identified for the main clinical outcomes, further multivariable meta-regression was not pursued.

**Nested Subgroup Analyses (BCID Alone vs. BCID+ASP Stratified by ASP Intensity):**

Nested subgroup analyses explored whether the comparative effect of BCID alone versus BCID+ASP differed within ASP-intensity strata. For time to appropriate therapy, matched within-stratum BCID alone versus BCID+ASP comparisons were not estimable within the remaining ASP-intensity strata. For length of stay, matched within-stratum BCID alone versus BCID+ASP comparisons were likewise not estimable within the remaining ASP-intensity strata. Overall, these nested analyses did not demonstrate statistically significant differences between BCID alone and BCID+ASP within the same ASP-intensity stratum.

**7. Results S4. Additional Time-to-Appropriate Therapy Findings**

To explore the substantial heterogeneity in the TTAT meta-analysis (I² = 81.8%), subgroup analyses by implementation context were performed. PCR BCID alone (3 studies) demonstrated a consistent TTAT reduction (MD −17.35 h, 95% CI −25.08 to −9.61; I² = 0%), while PCR BCID + ASP (4 studies) also showed significant reduction (MD −17.93 h, 95% CI −28.47 to −7.39; I² = 90.6%). The subgroup difference was not significant (χ² = 0.01, P = 0.92). Residual heterogeneity likely reflects unmeasured factors including ASP workflow responsiveness, implementation fidelity, and patient acuity.

**8. Results S5. Additional Mortality Findings**

Across individual studies, a small number reported statistically significant mortality differences, and the direction of effect was not uniform. For example, Bandy et al. (2023) reported that the combination of PCR BCID and ASP was associated with lower mortality (OR = 0.27, 95% CI: 0.09 to 0.75), whereas Roshdy et al. (2015) reported higher mortality (OR = 3.61, 95% CI: 1.19 to 10.89). However, these contrasting single-study findings appear to be exceptions within broader literature that, overall, does not demonstrate a statistically significant pooled mortality effect of PCR BCID implementation, including in studies conducted with and without concomitant ASP.

**9. Results S6. Additional Antimicrobial Stewardship Results**

Time to first appropriate de-escalation (3 studies; n = 815) was reduced by −15.24 hours (95% CI −20.60 to −9.89; P < 0.00001; I² = 48%), and time to first appropriate escalation (3 studies; n = 656) was reduced by −25.57 hours (95% CI −36.59 to −14.56; P < 0.00001; I² = 69%). The subgroup difference was significant (P = 0.006), indicating that BCID panels have differential effects on stewardship processes. Individual study results for de-escalation were consistently significant (Banerjee et al. −15.00 h, Gawrys et al. −23.90 h, Tseng et al. −10.70 h), whereas escalation effects were less consistent. These findings suggest BCID implementation most consistently accelerates appropriate de-escalation, with a less predictable but significant effect on escalation.

**10. Results S7. Additional Economic Results**

Clinical observations of total hospitalization costs (2 studies) revealed a statistically significant and substantial mean reduction of −$8,761 (95% CI: −$10,188 to −$3,334; P = 0.0001) with no heterogeneity (Tau² = 0.00, I² = 0%). Similarly, analysis of antimicrobial costs alone (2 studies) demonstrated a significant but smaller reduction of −$353 (95% CI: −$586 to −$120; P = 0.003), also with no heterogeneity (Tau² = 0.00, I² = 0%).

Among individual studies, Banerjee et al. (2015) showed the smallest total cost reduction (−$3,279), while Box et al. (2015) demonstrated consistent savings across both total hospitalization (−$6,906) and antimicrobial costs (−$342).

**11. Results S8. Publication Bias Assessment**

Evaluation of publication bias was conducted by means of Contour-Enhanced Funnel Plots, Egger’s Regression Test, and Begg’s Rank-Correlation Test.

Time to Appropriate Therapy: The funnel plot (Figure S1) showed symmetric distribution. Egger's test (intercept −0.97, P = 0.700) and Begg's test (tau = −0.14, P = 0.773) were non-significant. However, with k = 7 studies, these tests were underpowered.

Length of Stay: The funnel plot (Figure S2) did not suggest asymmetry. Egger's test (intercept −0.45, P = 0.334) and Begg's test (tau = −0.37, P = 0.098) were non-significant.

Mortality: Nineteen studies were included. The funnel plot (Figure S3) appeared symmetrically. Egger's test (intercept 0.41, P = 0.536) and Begg's test (tau = 0.04, P = 0.834) were non-significant. Trim-and-fill analysis did not materially change the pooled estimate.

Economic Outcomes: With only four estimates, formal testing was underpowered. Egger's test (intercept 6.97, P = 0.064) and Begg's test (tau = 1.00, P = 0.042) suggested possible asymmetry, but findings are unstable and should not be overinterpreted.

Overall, publication bias analyses supported stability of findings for TTAT and LOS, whereas economic estimates appeared more sensitive to between-study variability.

**12. Results S9. Temporal Stability Analysis**

A chronological cumulative meta-analysis examined how the results changed over time as new studies were added one at a time. For TTAT and LOS, the direction and magnitude of the pooled estimates remained stable across all stages of the cumulative sequence of studies. The Economic Outcomes results showed some variation early in the cumulative process (with Hedges' g ranging from -0.02 to +0.26) but ultimately yielded a non-significant overall pooled estimate. The Cumulative Effect for Stewardship Outcomes was initially favorable; however, it was reduced with each additional study. Finally, the Mortality Estimate changed over time, and while the estimate fluctuated, no large directional reversals occurred. Overall, temporal analyses supported stability of findings for time to appropriate therapy and length of stay, whereas economic and stewardship estimates were more sensitive to study accumulation.

**13. Results S10. Prediction Intervals and Trial Sequential Analysis**

Prediction intervals provided additional context for the pooled estimates across heterogeneous implementation settings. For time to appropriate therapy, the pooled mean difference was −17.28 hours, with a 95% prediction interval of −33.96 to −0.60 hours. For length of stay, the pooled mean difference was −1.25 days, with a 95% prediction interval of −2.29 to −0.22 days. These intervals remained on the side favoring BCID implementation. In contrast, the mortality prediction interval crossed the null, indicating that future similar studies could remain compatible with benefit, no effect, or harm.

Trial sequential analysis supported shorter time to appropriate therapy and shorter length of stay. For time to appropriate therapy, using a prespecified clinically important reduction of 6 hours, the heterogeneity-adjusted DARIS was 1533 and the accrued sample size was 1453; the cumulative Z-curve crossed the monitoring boundary. For length of stay, using a prespecified clinically important reduction of 1 day, the heterogeneity-adjusted DARIS was 2462 and the accrued sample size was 3037; the cumulative Z-curve crossed the monitoring boundary and accrued information exceeded DARIS. Mortality remained inconclusive. Using a 20% relative risk reduction, the DARIS was 3588 and the accrued sample size was 2987; the cumulative Z-curve crossed neither the efficacy nor harm boundary.

**13. S11. SUPPLEMENTARY FIGURES**

**Supplementary Figure S1. Contour-Enhanced Funnel Plot for Time to Appropriate Therapy**
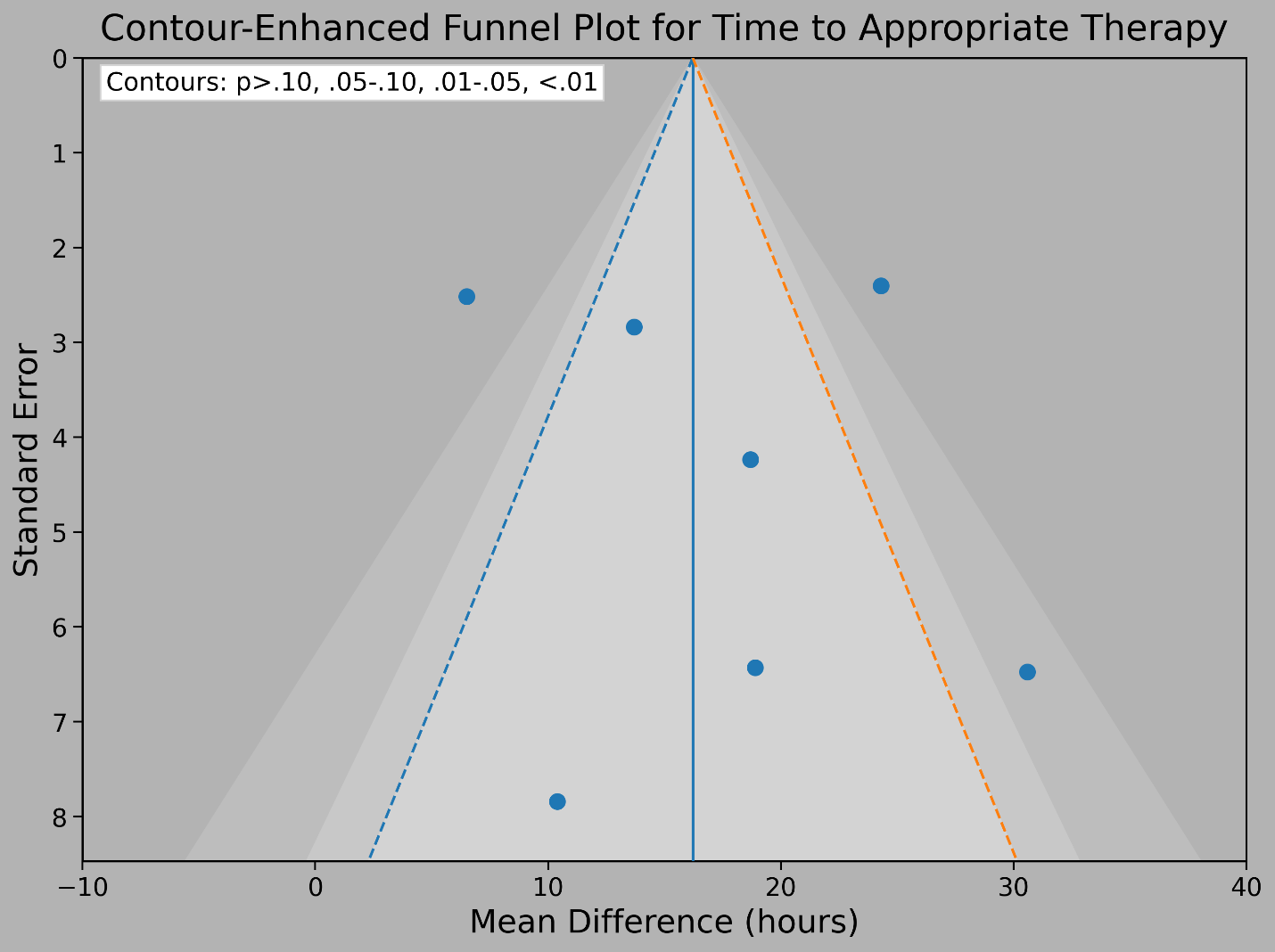


Contour-enhanced funnel plot assessing publication bias for time-to-appropriate therapy. The x-axis represents the effect size (mean difference in hours), and the y-axis represents the standard error. The vertical line indicates the pooled effect estimate, and the diagonal lines represent the pseudo 95% confidence limits. Shaded significance contours indicate conventional significance regions and help distinguish whether any asymmetry is more compatible with publication bias or with other small-study effects.

**Supplementary Figure S2. Contour-Enhanced Funnel Plot for Length of Stay**

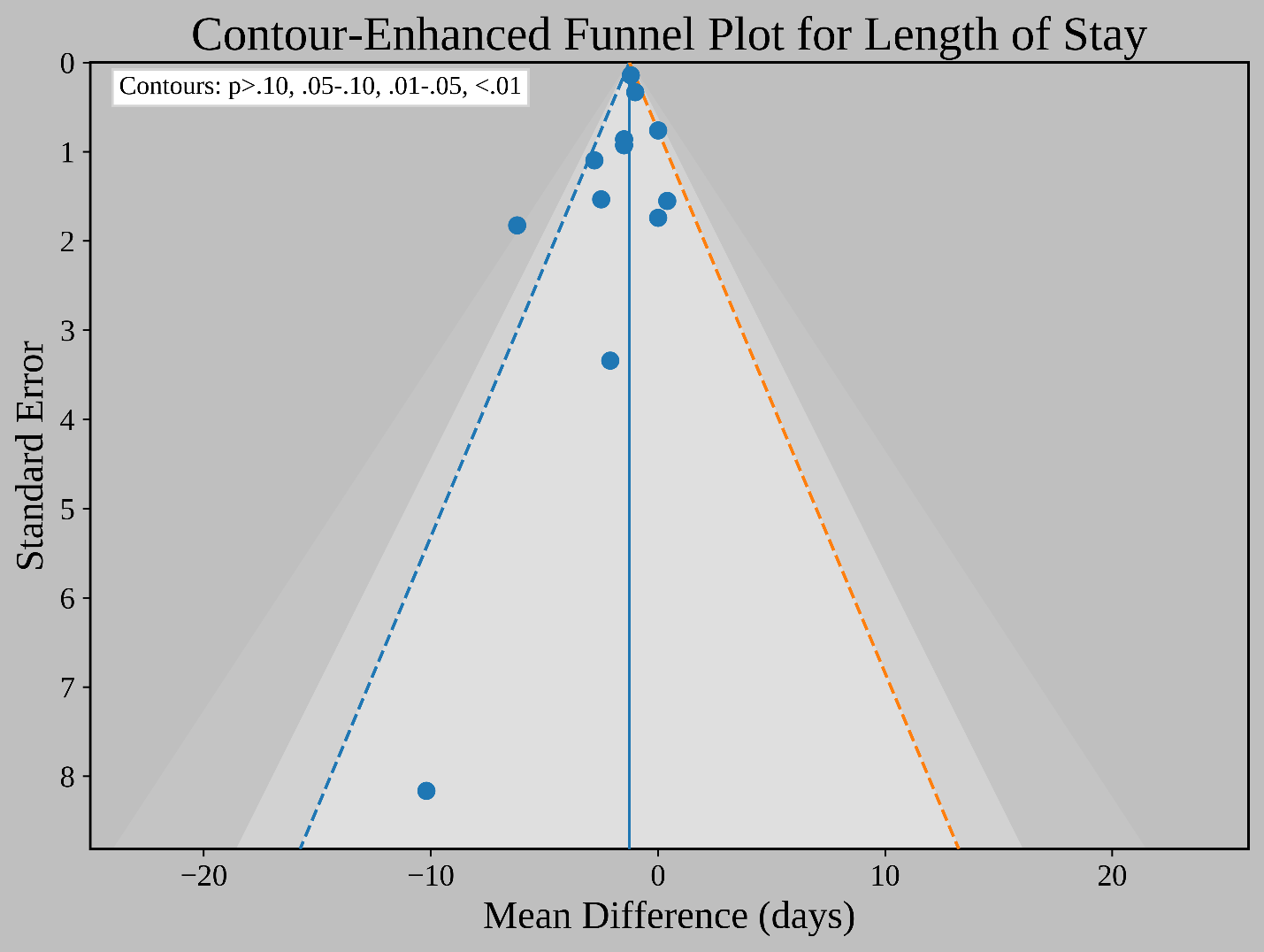


Contour-enhanced funnel plot assessing publication bias for length of stay. The x-axis represents the effect size (mean difference in days), and the y-axis represents the standard error. The vertical line indicates the pooled effect estimate, and the diagonal lines represent the pseudo 95% confidence limits. Shaded significance contours indicate conventional significance regions and were added to help distinguish possible publication bias from alternative causes of funnel-plot asymmetry.

**Supplementary Figure S3. Contour-Enhanced Funnel Plot for Mortality**


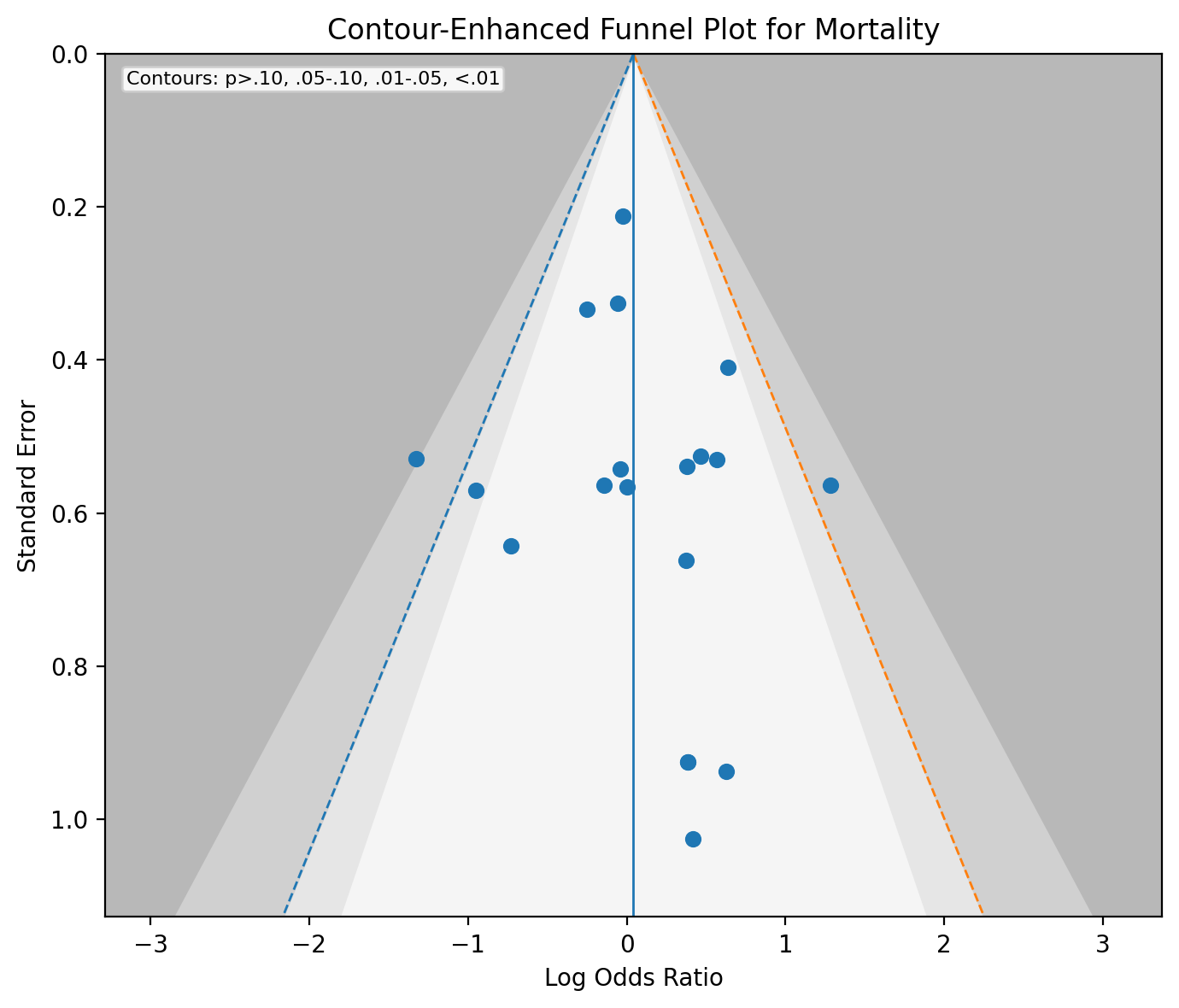

Contour-enhanced funnel plot assessing publication bias for mortality. The x-axis represents the effect size (log odds ratio), and the y-axis represents the standard error. The vertical line indicates the pooled effect estimate, and the diagonal lines represent the pseudo 95% confidence limits. Shaded significance contours indicate conventional significance regions and help assess whether any asymmetry is more suggestive of publication bias or other small-study effects. Visual inspection suggests a broad symmetric distribution, although power remains limited given the modest number of studies.

**Supplementary Figure S4. Risk of Bias Traffic Light Plot**


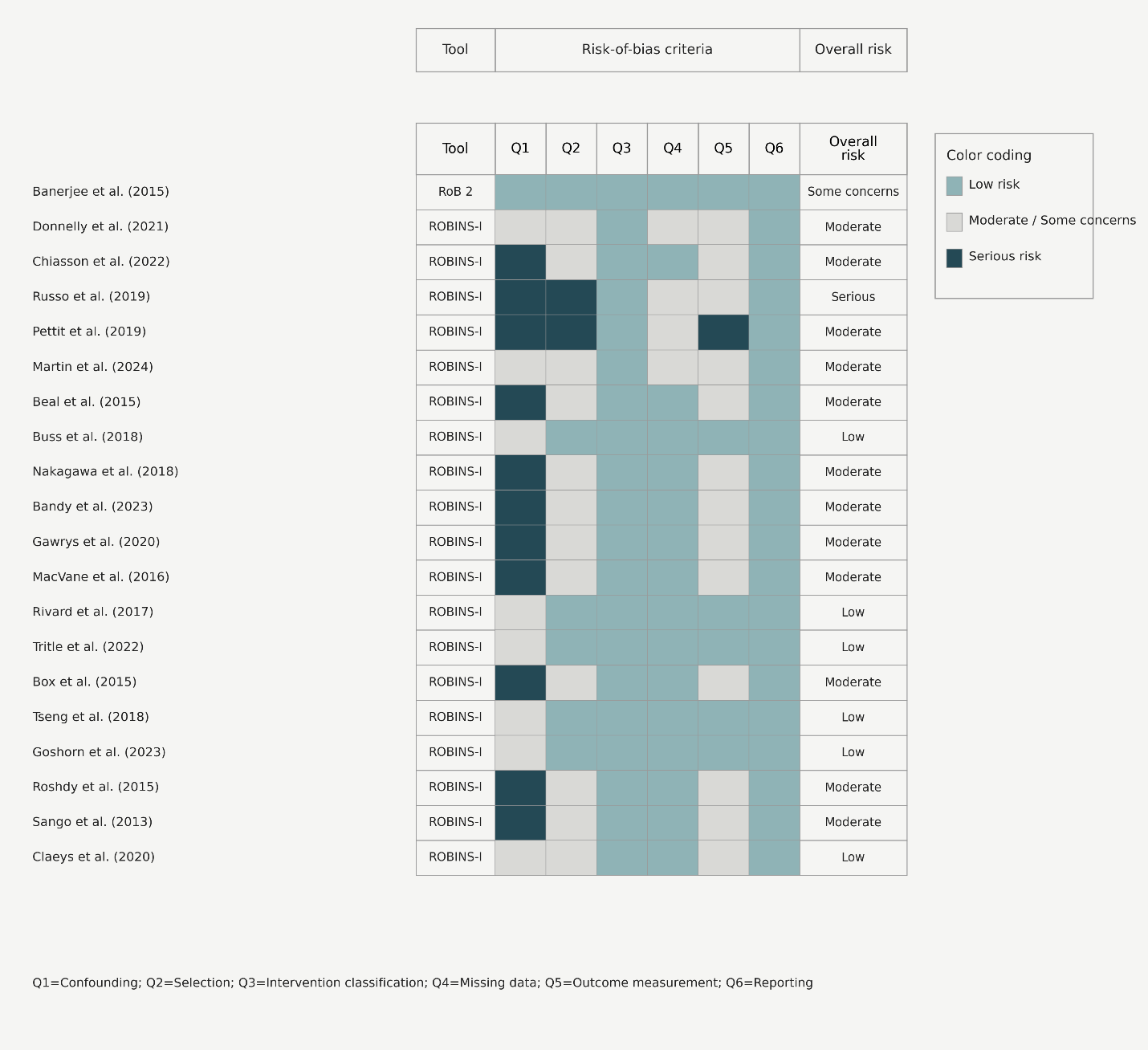


Study-level risk of bias across included studies. Banerjee et al. (2015) was assessed using RoB 2, whereas non-randomized studies were assessed using ROBINS-I. Columns represent the following domains: D1, confounding; D2, selection of participants; D3, classification of interventions; D4, missing data; D5, outcome measurement; and D6, selection of reported results. Cell shading indicates the judged risk level for each domain (green = low risk; yellow = moderate risk/some concerns; red = serious/critical risk), and the final column shows the overall risk-of-bias judgment for each study.

**Supplementary Figure S5. Risk of Bias Summary Graph**


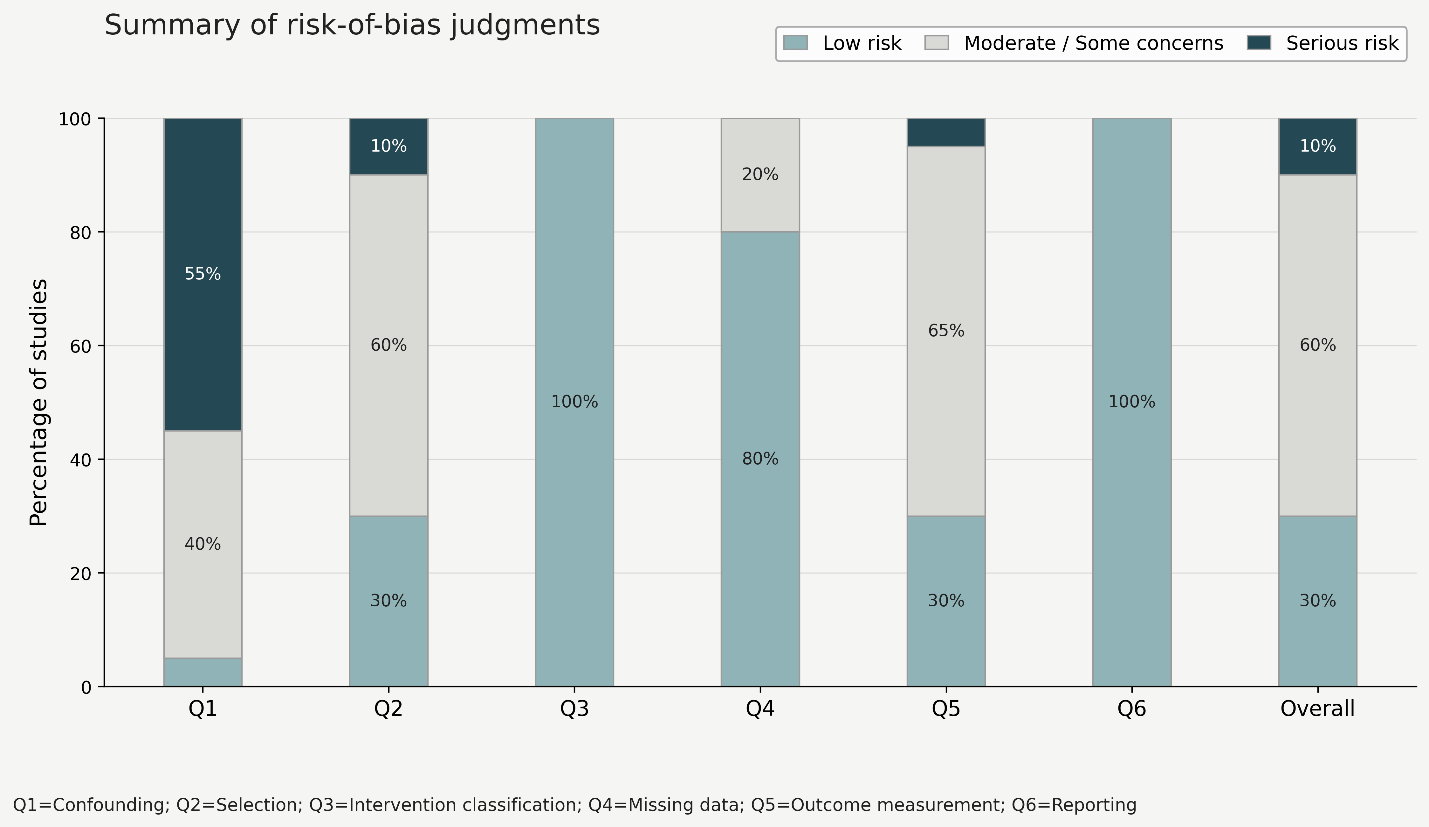


Stacked bar chart showing the proportion of included studies classified at each risk-of-bias level across the ROBINS-I domains and for the overall risk-of-bias judgment. Dark shading indicates lower risk, and lighter shading indicates higher risk. The graph summarizes the distribution of study-level bias concerns across the evidence base and highlights domains contributing most to methodological limitations.

**Supplementary Figure S6. Baujat Plots**


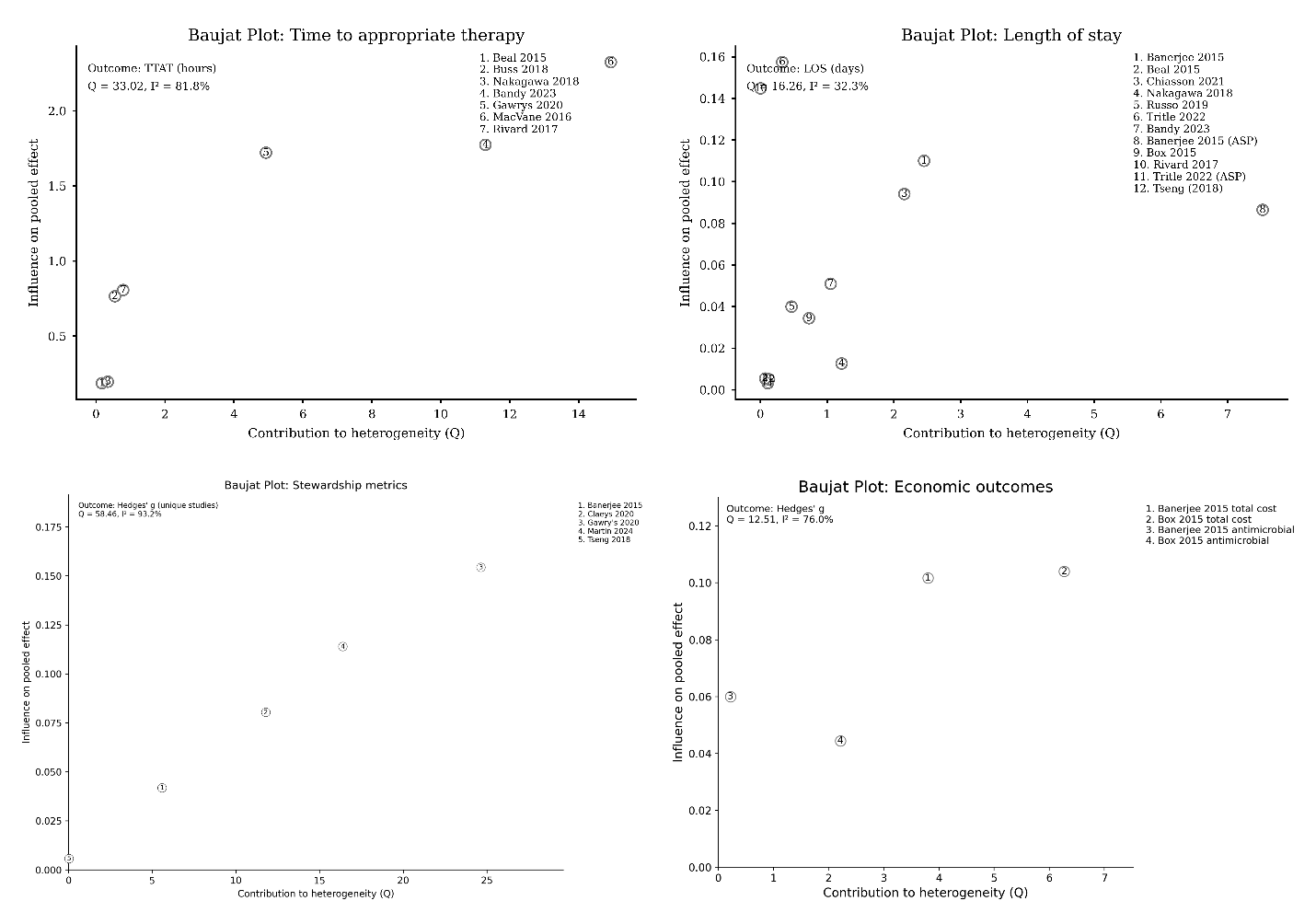


Baujat plots for (A) time to appropriate therapy, (B) length of stay, (C) Stewardship, and (D) economic outcomes. The x-axis represents each study's contribution to the heterogeneity statistic (Q), while the y-axis reflects its influence on the pooled estimate. Studies located toward the upper-right portion of the plot have the greatest combined impact on inconsistency and on the summary effect size.

**Supplementary Figure S7. Cumulative Meta-Analysis**


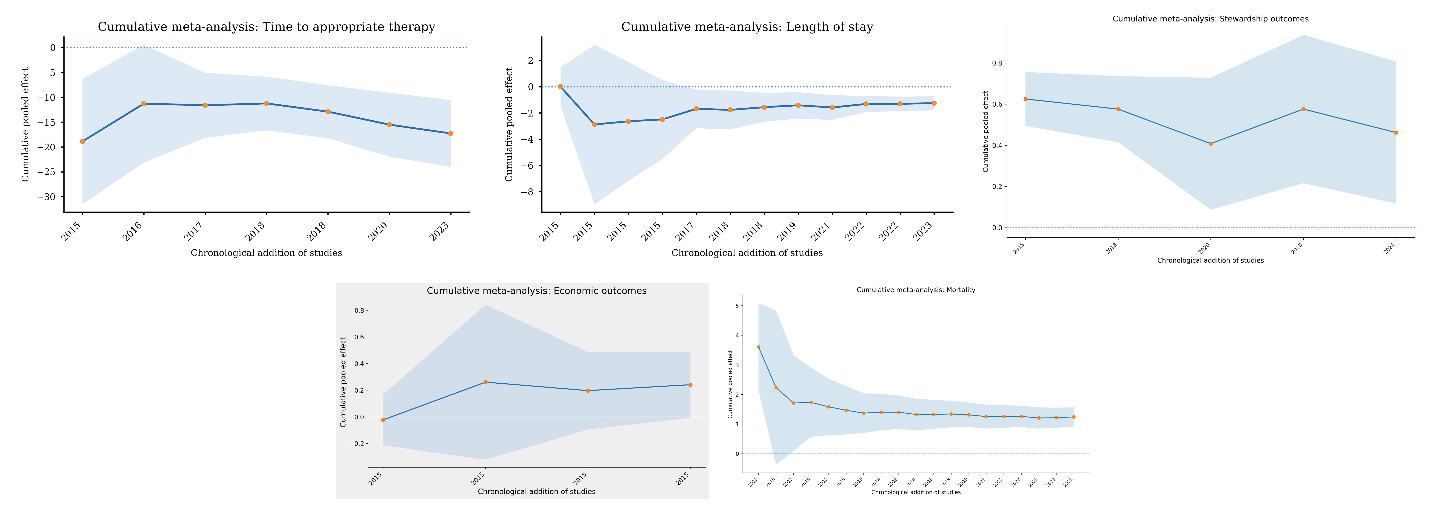


Cumulative meta-analysis plots (A) TTAT, (B) LOS, (C) stewardship outcomes (D) economic outcomes, and (E) Mortality, showing the evolution of pooled estimates as studies were added chronologically by publication year. The x-axis represents the cumulative pooled effect estimate, and the y-axis lists studies in order of publication.

**Supplementary Figure S8. Time to appropriate therapy trial sequential analysis.**

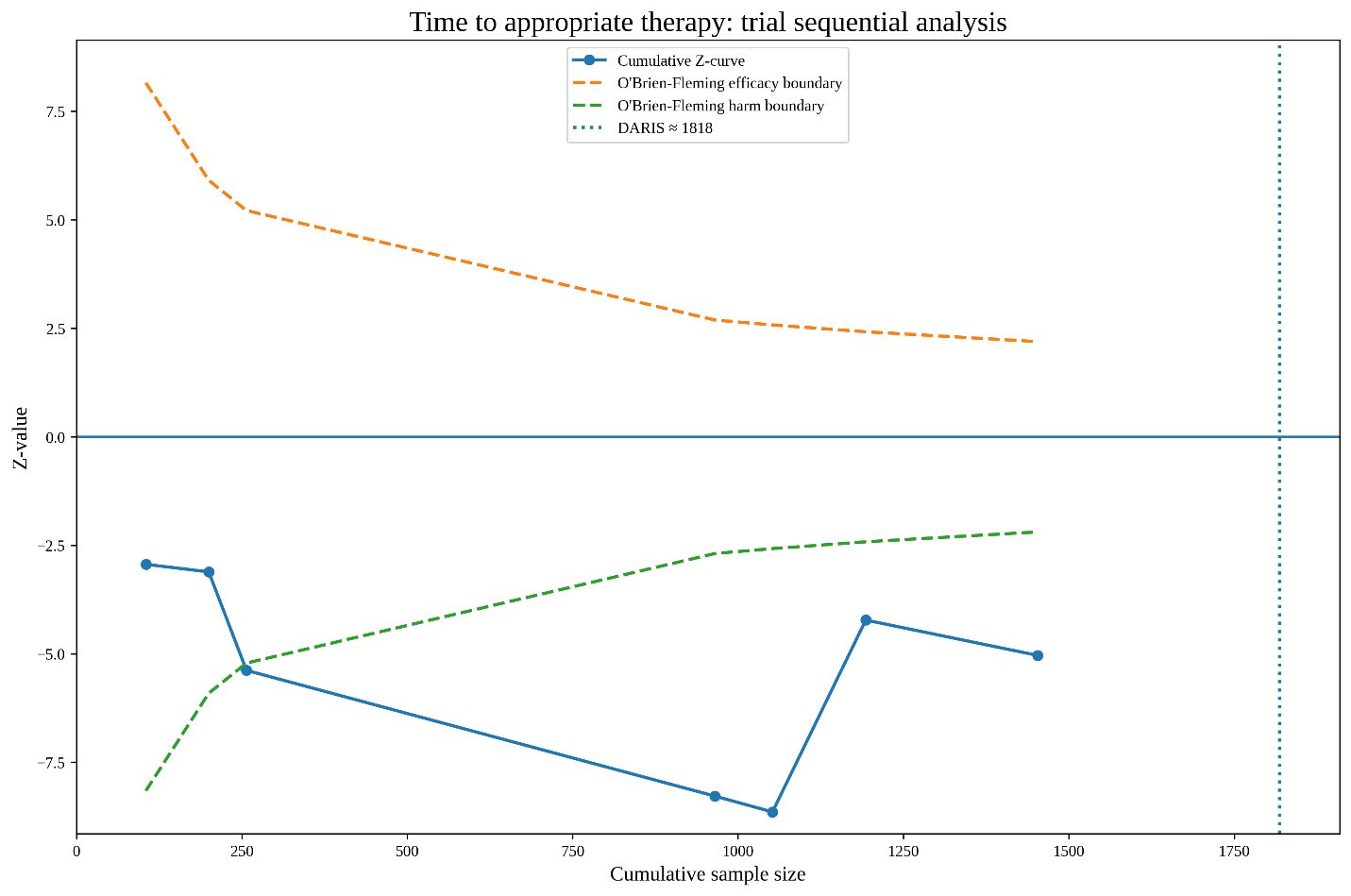


Cumulative Z-curve for time to appropriate therapy compared with O’Brien-Fleming monitoring boundaries. The heterogeneity-adjusted required information size was 1818 participants, based on a prespecified clinically important reduction of 6 hours.

**Supplementary Figure S9. Length of stay trial sequential analysis.**


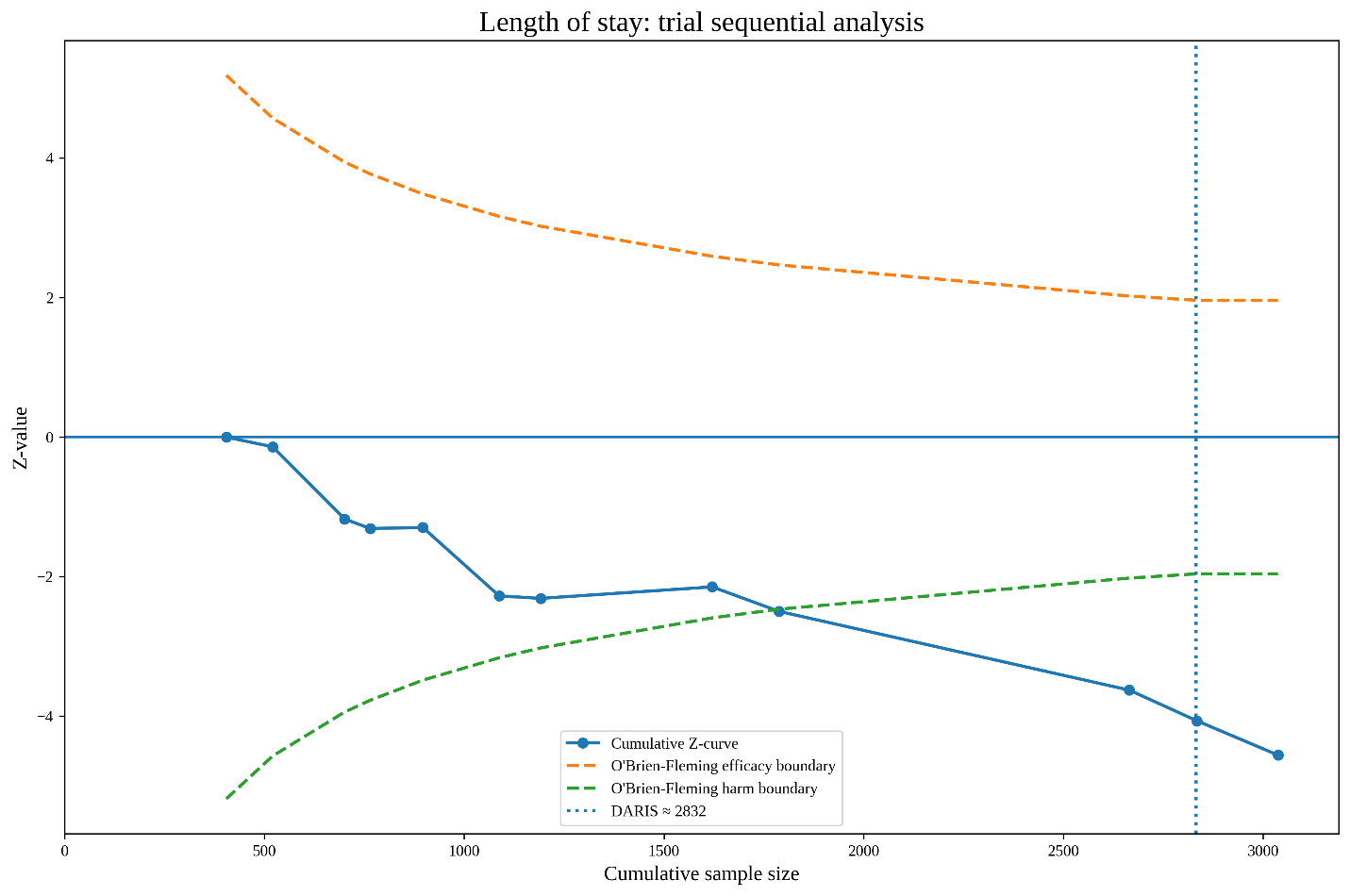
 Cumulative Z-curve for hospital length of stay compared with O’Brien-Fleming monitoring boundaries. The heterogeneity-adjusted required information size was 2832 participants, based on a prespecified clinically important reduction of 1 day.

**Supplementary Figure S10. Mortality trial sequential analysis.**

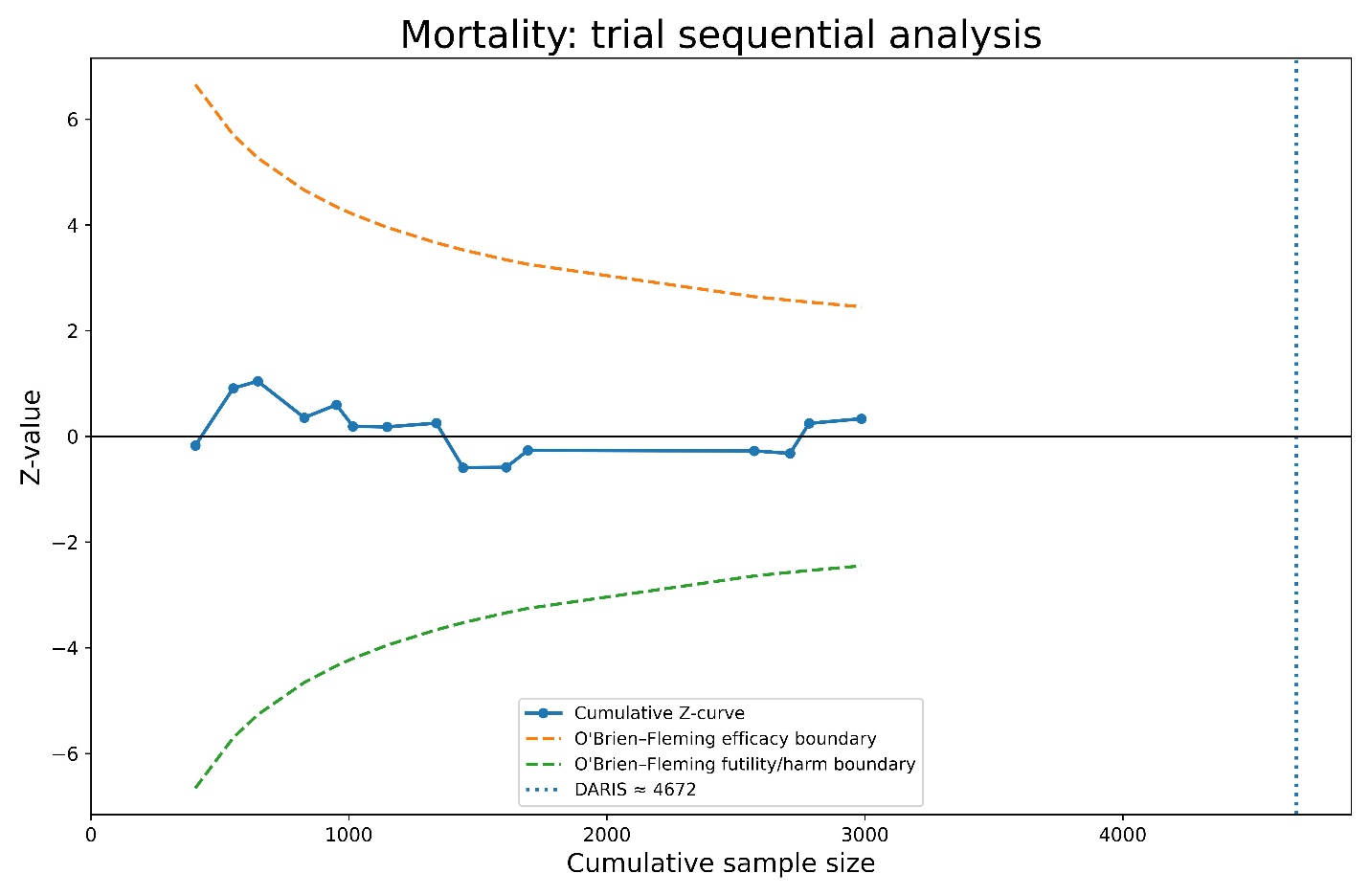
 Cumulative Z-curve for mortality compared with O’Brien-Fleming monitoring boundaries. The heterogeneity-adjusted required information size was 4672.2 participants, based on an assumed 20% relative risk reduction.

**14. Discussion S1. Extended Economic Discussion**

The primary basis for interpreting the findings of the economic studies will be the direct clinical cost analysis conducted in the pooled estimates. In addition to demonstrating savings across both total hospitalization and antimicrobial costs (Box et al., 2015), Banerjee et al. (2015) also showed less clear-cut total cost differences when comparing the two groups, however the authors did show significant stewardship gains that likely would support a subsequent economic benefit. When viewed as a whole, this evidence suggests that the best way to demonstrate the value of PCR-based bloodstream culture identification is through the reduction of hospital-based utilization and targeted use of antibiotics in patients, rather than through economic models used outside of the patient population.

**Discussion S2. Extended TTAT Heterogeneity and Meta-Regression Discussion**

Exploratory Meta-Regression to Interpret Heterogeneity:

Meta-regression analysis identified substantial residual heterogeneity in results from studies evaluating TTAT. The results indicate that BCID Panels used in Antimicrobial Stewardship Programs (ASPs) have an important impact on optimizing time to initiate Antimicrobial Therapy. However, the magnitude of benefit across implementation strategies and ASP-intensity categories was variable, and meta-regression did not identify a statistically significant contribution of these factors to the observed heterogeneity.
The residual heterogeneity suggests that the pooled effect is an average across a large number of implementation settings that differ substantially from one another. Both the BCID-Alone and BCID+ASP studies demonstrated a reduction in TTAT; however, the subgroup comparison was not statistically significant. This may suggest that the benefits of using either strategy are favorable for reducing time-to-antibiotic therapy, regardless of how they are implemented. However, the magnitude of the reduction may still depend on the specific implementation context of each study.
Many variables could have contributed to the variability observed in this study. Variables that were not measured included workflow for the stewardship team at each site, how many pharmacists were available, how quickly clinicians responded to the stewardship team's requests, the level of acuity of patients at each site, and the definition of what was considered a "appropriate" therapy as defined by the stewardship team. The variation observed in this study is representative of how different sites may integrate the BCID (and similar tools) into their workflows, rather than simply reflecting random sampling error. Future research should better describe the site-level contextual issues related to BCID integration so that subsequent researchers can understand which contexts yield the greatest benefit from using these tools.

**Discussion S3. Extended Mortality Interpretation**

There is a disconnect between the goals of process-oriented endpoints and those of outcome-oriented endpoints, suggesting that translating advancements in microbiologic diagnostics into lower mortality in patients with bloodstream infection can be difficult. In addition to the choice of antibiotics and when they should be administered, mortality from bloodstream infections is influenced by many other variables, including host immune function, adequacy of source control, degree of illness at the time of presentation, timeliness of sepsis recognition, and organ support.

Moreover, because BCID testing is typically performed only after blood cultures signal positivity, the intervention occurs after an initial period in which outcomes in the sickest patients may already be strongly determined. As a result, even meaningful gains in time to organism identification and optimization of therapy may be insufficient to shift survival in many real-world settings.

In the present analysis, PCR-based BCID panels were not associated with a statistically significant reduction in mortality, including in studies conducted with and without concomitant antimicrobial stewardship. This suggests that rapid diagnostic information—even when acted upon—may not fully overcome preexisting pathophysiologic and time-dependent constraints that drive mortality in severe bloodstream infections. Overall, while PCR-based BCID panels represent an important tool to improve the accuracy and efficiency of antimicrobial management and are likely to support stewardship interventions and reductions in hospital length of stay, current evidence does not support a direct survival benefit.

**Discussion S4. Extended Antimicrobial Stewardship Narrative**

The meta-analysis demonstrates significant improvements in stewardship response times: a 15.24-hour decrease in time to de-escalation and 25.57-hour decrease in time to escalation. These data also demonstrate the dual purpose of BCID: the ability to quickly provide narrow-spectrum treatment for likely causative organisms, while rapidly providing a broad-spectrum treatment if resistant organisms are identified. For gram-positive infections, the panels optimize the treatment of methicillin-sensitive Staphylococcus aureus (MSSA) and vancomycin-resistant enterococci (VRE). For gram-negative infections, they allow immediate identification of resistance, enabling appropriate escalation of the antibiotic regimen.

In addition, by allowing early discontinuation of unnecessary antibiotics, these systems can decrease adverse drug events, *Clostridioides difficile* infections, and antimicrobial resistance. The data indicate earlier de-escalation of anti-methicillin-resistant Staphylococcus aureus (MRSA) and anti-pseudomonal agents, suggesting that BCID panels will help Antibiotic Stewardship Programs (ASPs) limit their use of broad-spectrum antibiotics.

BCID panels and ASP have a complementary relationship: diagnostics generate rapid, accurate data, whereas stewardship enables clinicians to interpret these data to guide appropriate clinical decision-making. Martin et al. (2024) also found that when ASP support is provided to BCID panels, the greatest benefit will be realized from using these panels. When used in conjunction with comprehensive stewardship programs, the ability of BCID technology to optimize therapy in a timely manner and limit unnecessary antibiotic use is maximized.

**Discussion S5. Strengths of This Review**

This systematic review has many strengths. First, by deliberately focusing on U.S. health care delivery systems, the results of this review are directly applicable to domestic hospital decision-makers contemplating the adoption of BCID. Second, the wide-ranging nature of the outcome measures assessed in this review — that is, time to appropriate antibiotic therapy; in-hospital mortality; length of hospital stay; stewardship metrics such as rates of vancomycin-resistant Enterococcus (VRE); and costs — provides an integrated overview of the overall impact of BCID. Third, the use of advanced statistical methods, including random-effects models for estimating pooled effects and variance components, and a ROBINS-I/RoB 2 assessment of study risk of bias, in addition to numerous sensitivity analyses (that is, leave-one-out, Hartung-Knapp, meta-regression, influence diagnostic analyses), increases the level of confidence in the pooled estimates. Fourth, the pre-specification of subgroup analyses based on the degree of Antimicrobial Stewardship Program (ASP) intensity, pathogen-specificity, and platform type guides practical implementation considerations.

**Discussion S6. Policy Implications and Future Research Directions**

**Policy Implications:**

Hospital administration should consider BCID an investment in the overall quality of hospital care rather than a laboratory cost, with the potential to reduce patient lengths of stay and optimize resources. The subgroup analysis comparing outcomes of patients treated with BCID-only and BCID + ASP did not show statistical significance. However, there is some evidence to suggest that hospital administrators implement BCID panels as part of a comprehensive stewardship program that includes pharmacists, a real-time notification system, and clinical staff education.

**Future Research Directions:**

Rapidly developing technologies have the potential to solve existing problems with BCIDs. Whole-blood tests that do not require culture could allow for results within 4 hours; Iyer et al. (2024) used the RaPID/BSI test to identify 102 pathogens, while conventional culture methods identified 54 pathogens in the same samples. This technology could also reduce the 12-hour mortality rate that is associated with slow BCIDs. Although metagenomics-based assays can detect a wide variety of pathogens, they are still in early stages of development and face several challenges. Challenges include high costs and difficulty distinguishing between pathogens and contaminations.

Most importantly, there is an urgent need for large randomized controlled trials to show a mortality benefit of faster BCIDs. In fact, the ASM 2025 Guidelines stated that there is limited evidence supporting the use of BCIDs for diagnosing bacterial infections, due to a lack of large-scale randomized controlled trials. Future studies will be needed to evaluate how to implement this design in clinical practice. They will need to include evaluation of long-term outcome measures, including antimicrobial resistance rates and *Clostridioides difficile* incidence.

**Alt Text**

**Supplementary Figure S1.** Contour-enhanced funnel plot for time to appropriate therapy, showing study effect estimates against standard errors with the pooled effect estimate and pseudo 95% confidence limits.

**Supplementary Figure S2.** Contour-enhanced funnel plot for length of stay, showing study effect estimates against standard errors with the pooled effect estimate and pseudo 95% confidence limits.

**Supplementary Figure S3.** Contour-enhanced funnel plot for mortality, showing log odds ratios against standard errors with the pooled effect estimate and pseudo 95% confidence limits.

**Supplementary Figure S4.** Risk-of-bias traffic light plot showing study-level judgments across ROBINS-I domains for non-randomized studies and RoB 2 domains for the randomized trial.

**Supplementary Figure S5.** Risk-of-bias summary graph showing the proportion of included studies classified at each risk-of-bias level across domains and overall judgments.

**Supplementary Figure S6.** Baujat plots showing each study’s contribution to heterogeneity and influence on pooled effect estimates for time to appropriate therapy, length of stay, stewardship outcomes, and economic outcomes.

**Supplementary Figure S7.** Cumulative meta-analysis plots showing how pooled estimates changed as studies were added chronologically for time to appropriate therapy, length of stay, stewardship outcomes, economic outcomes, and mortality.

**Supplementary Figure S8.** Trial sequential analysis for time to appropriate therapy showing the cumulative Z-curve compared with O’Brien-Fleming monitoring boundaries and the required information size.

**Supplementary Figure S9.** Trial sequential analysis for hospital length of stay showing the cumulative Z-curve compared with O’Brien-Fleming monitoring boundaries and the required information size.

**Supplementary Figure S10.** Trial sequential analysis for mortality showing the cumulative Z-curve compared with O’Brien-Fleming efficacy and harm boundaries and the required information size.
