# Supplementary material for: Impact of Multiplex PCR Blood-Culture Identification Panels on Clinical Outcomes, Antimicrobial Stewardship, and Economic Impact in US Hospitals: A Systematic Review and Meta-Analysis
Source: Open Forum Infect Dis. 2026 Jun 25;13(7):ofag370. doi: 10.1093/ofid/ofag370 (PMC13332404; doi:10.1093/ofid/ofag370)
Supplement: ofag370_Supplementary_Data [file ofag370_supplementary_data.zip › Supplementary_Table_OFID_revised May 27-2026.docx]

**Supplementary Table S1. Summary of Sensitivity and Exploratory Analyses**

**A. Leave-One-Out Sensitivity Analyses**

| **Outcome** | **Study Excluded** | **Effect on Pooled Estimate** | **Effect on Heterogeneity (I²)** | **Interpretation** |
| --- | --- | --- | --- | --- |
| Time to appropriate therapy | MacVane et al. (2016) | MD remained significant | 81.8% → 59.8% | Principal contributor to heterogeneity; pooled effect robust |
| Time to appropriate therapy | All others sequentially | MD remained significant and directionally consistent | Minimal to moderate change | No single study drove the result |
| Length of stay | Banerjee et al. (2015) | MD remained significant | 32.3% → 0.0% | Principal contributor to heterogeneity; pooled effect robust |
| Length of stay | All others sequentially | MD remained significant and directionally consistent | Minimal change | No single study drove the result |
| Economic outcomes | Box et al. (2015) | Hedges' g 0.14 (95% CI −0.07 to 0.35); p=0.20 | 75.8% → 61.8% | Principal contributor: effect remained non-significant |
| Stewardship outcomes | Gawrys et al. (2020) | Effect remained favorable | 93.2% → 90.2% | Heterogeneity remained substantial |

**B. Hartung–Knapp Reanalysis**

| **Outcome** | **DerSimonian-Laird Estimate** | **Hartung–Knapp Estimate** | **P-value (H-K)** | **Interpretation** |
| --- | --- | --- | --- | --- |
| Time to appropriate therapy | MD −17.28 h (95% CI −24.00 to −10.56) | MD −17.28 h (95% CI −24.81 to −9.75) | p=0.0014 | Remained significant |
| Length of stay | MD −1.25 d (95% CI −1.79 to −0.71) | MD −1.25 d (95% CI −1.95 to −0.56) | p=0.0021 | Remained significant |
| Economic outcomes | Hedges’ g 0.24 (95% CI −0.18 to 0.67) | Hedges’ g 0.24 (95% CI −0.16 to 0.64) | p=0.15 | Remained non-significant |
| Stewardship outcomes | Hedges' g 0.46 (95% CI 0.24 to 0.68) | Hedges' g 0.46 (95% CI −0.07 to 1.00) | p=0.07 | Attenuated; no longer significant |

**C. Fixed-Effect vs. Random-Effects Model Comparison**

| **Outcome** | **Fixed-Effect Estimate** | **Random-Effects Estimate** | **Interpretation** |
| --- | --- | --- | --- |
| Time to appropriate therapy | MD -16.22 h (95% CI -18.80 to -13.64) | MD -17.28 h (95% CI -24.00 to -10.56) | Consistent across models |
| Length of stay | MD -1.19 d (95% CI -1.43 to -0.95) | MD -1.25 d (95% CI -1.79 to -0.71) | Consistent across models |
| Mortality | Similar direction and significance | OR 1.04 (95% CI 0.81–1.34) | Consistent across models |
| Economic outcomes | Narrower CI; borderline | Hedges’ g 0.24 (95% CI −0.18 to 0.67) | Sensitive to model choice; random-effects non-significant |
| Stewardship outcomes | Similar direction and significance | Hedges' g 0.46 (95% CI 0.24 to 0.68) | Consistent across models |

**D1. Subgroup Analyses by ASP Intensity**

| **Outcome** | **Low Intensity (k)** | **Moderate Intensity (k)** | **High Intensity (k)** | **Test for Subgroup Difference** | **Heterogeneity Explained (R²)** |
| --- | --- | --- | --- | --- | --- |
| Time to appropriate therapy | −15.48 h; I²=0% (k=2) | −18.70 h; I²=0% (k=1) | −17.93 h; I²=90.6% (k=4) | χ²=0.39, p=0.82 | 0% |
| Length of stay | −0.10 d; I²=0% (k=2) | −1.37 d; I²=27.4% (k=4) | −1.61 d; I²=47.0% (k=6) | χ²=2.32, p=0.31 | 6.1% |

**D2. Subgroup Analyses by Implementation Model**

| **Outcome** | **BCID Alone (k)** | **BCID+ASP (k)** | **Test for Subgroup Difference** | **Interpretation** |
| --- | --- | --- | --- | --- |
| Time to appropriate therapy | −17.35 h; I²=0% (k=3) | −17.93 h; I²=90.6% (k=4) | Not significant | No significant effect modification |
| Length of stay | −0.99 d; I²=18.4% (k=6) | −1.61 d; I²=47.0% (k=6) | Not significant | No significant effect modification |

**E. Subgroup Analyses by Pathogen Specificity**

| **Outcome** | **Broad/Unspecified (k)** | **Gram-Negative (k)** | **Gram-Positive (k)** | **Test for Subgroup Difference** | **Heterogeneity Explained** |
| --- | --- | --- | --- | --- | --- |
| Time to appropriate therapy | −6.86 h; I²=0% (k=2) | −21.15 h; I²=82.5% (k=2) | −22.55 h; I²=0% (k=3) | χ²=25.73, p<0.0001 | 68.5% |
| Length of stay | −1.57 d; I²=60.6% (k=6) | −1.20 d (k=1) | −0.97 d; I²=0% (k=5) | χ²=0.07, p=0.97 | 0% |
| Mortality | OR 1.22; I²=0% (k=12) | OR 0.98 (k=1) | OR 1.36; I²=64% (k=6) | χ²=0.55, p=0.76 | 3.0% |

**F. Subgroup Analyses by BCID Platform Type**

| **Outcome** | **BioFire (k)** | **Verigene (k)** | **ePlex (k)** | **Test for Subgroup Difference** | **Heterogeneity Explained** |
| --- | --- | --- | --- | --- | --- |
| Time to appropriate therapy | −7.39 h; I²=0% (k=2) | −21.41 h; I²=80.6% (k=5) | — | χ²=9.03, p=0.003 | 30.2% |
| Length of stay | −1.44 d; I²=54.4% (k=7) | −1.20 d; I²=0% (k=5) | — | χ²=0.03, p=0.87 | 0.2% |
| Mortality | OR 1.08; I²=0% (k=9) | OR 1.29; I²=58% (k=7) | OR 1.61; I²=0% (k=2) | χ²=0.91, p=0.64 | 5.1% |

Platform subgroup analyses were feasible primarily for TTAT, LOS, and mortality. For stewardship and economic outcomes, platform-based subgroup comparisons were limited by sparse data and did not show significant effect modification.

**G. Univariable Meta-Regression**

| **Outcome** | **Covariate** | **β Coefficient (95% CI)** | **P-value** | **R² (Heterogeneity Explained)** |
| --- | --- | --- | --- | --- |
| Time to appropriate therapy | ASP intensity score | −0.99 (−8.94 to 6.96) | 0.807 | 0% |
| Time to appropriate therapy | Implementation model | 5.85 (−4.78 to 16.47) | 0.281 | 32.6% |
| Length of stay | ASP intensity score | −0.54 (−1.43 to 0.35) | 0.233 | 0% |
| Length of stay | Implementation model | −0.55 (−1.92 to 0.82) | 0.432 | 0% |
| Mortality | Implementation model | 0.02 (−0.49 to 0.52) | 0.948 | 24.3% |
| Economic outcomes | Cost category | 0.01 (−0.49 to 0.51) | 0.970 | Not significant |
| Stewardship outcomes | Stewardship subtype (de-escalation) | 0.57 (0.05 to 1.08) | 0.032 | 54.3% |
| Stewardship outcomes | Stewardship subtype (escalation) | 0.74 (0.22 to 1.27) | 0.006 | — |
| Length of stay | Multivariable model (ASP intensity + implementation model) | ASP −1.19 (−3.52 to 1.15); Impl 0.97 (−2.36 to 4.30) | 0.320 / 0.569 | **0.0% (model p=0.452)** |

**H. Risk of Bias Sensitivity Analyses**

| **Analysis Type** | **Outcome** | **Result** | **Interpretation** |
| --- | --- | --- | --- |
| Meta-regression (continuous NOS-equivalent score) | Time to appropriate therapy | β=3.26 (95% CI −4.34 to 10.85); p=0.401 | Quality score not a significant predictor |
| Meta-regression (continuous NOS-equivalent score) | Length of stay | β=0.06 (95% CI −0.56 to 0.67); p=0.859 | Quality score not a significant predictor |
| Meta-regression (continuous NOS-equivalent score) | Mortality | β=0.09 (95% CI −0.14 to 0.33); p=0.437 | Quality score not a significant predictor |
| Meta-regression (continuous NOS-equivalent score) | Economic outcomes | β=−0.44 (95% CI −0.94 to 0.06); p=0.085 | Trend toward association; not significant |
| Meta-regression (continuous NOS-equivalent score) | Stewardship outcomes | β=−0.20 (95% CI −0.54 to 0.14); p=0.246 | Quality score not a significant predictor |
| Categorical ROB moderator | All outcomes | No significant associations | Overall, ROB category did not predict effect size |
| Categorical ROB moderator | Time to appropriate therapy | No significant associations (P=0.211) | Overall, ROB category did not predict effect size |
| Categorical ROB moderator | Length of stay | No significant associations (P=0.613) | Overall, ROB category did not predict effect size |
| Domain-specific (confounding) | All outcomes | No significant effect modification | Confounding risk did not explain heterogeneity |
| Domain-specific (selection) | All outcomes | No significant effect modification | Selection risk did not explain heterogeneity |
| Domain-specific (outcome measurement) | All outcomes | No significant effect modification | Measurement risk did not explain heterogeneity |
| Quality-effects model | Time to appropriate therapy | Hedges' g 0.70 (95% CI 0.57 to 0.83); p<0.001 | Remained significant |
| Quality-effects model | Length of stay | Hedges' g 0.33 (95% CI 0.24 to 0.41); p<0.001 | Remained significant |
| Quality-effects model | Mortality | Log OR 0.04 (95% CI −0.20 to 0.28); p=0.75 | Remained non-significant |
| Quality-effects model | Economic outcomes | Hedges' g 0.15 (95% CI 0.02 to 0.29); p=0.024 | Modest favorable effect |

1. **Additional Sensitivity Analyses**

| **Analysis** | **Outcome** | **Result** | **Interpretation** |
| --- | --- | --- | --- |
| Median-to-mean conversion exclusion (Beal et al. (2015)) | Time to appropriate therapy | MD −17.10 h (95% CI −24.53 to −9.66); p<0.001 | Robust; not driven by converted statistics |
| Median-to-mean conversion exclusion | Length of stay | MD −1.26 d (95% CI −1.83 to −0.69); p<0.001 | Robust; not driven by converted statistics |
| Quality-effects model | Time to appropriate therapy | Hedges' g 0.70 (95% CI 0.57 to 0.83); p<0.001 | Remained significant |
| Quality-effects model | Length of stay | Hedges' g 0.33 (95% CI 0.24 to 0.41); p<0.001 | Remained significant |
| Prospective vs. retrospective design | Mortality | Prospective (k=18): OR 1.23; Retrospective (k=1): OR 1.60; p=0.61 | Study design did not explain heterogeneity |
| Cumulative meta-analysis | Time to appropriate therapy | Effect remained directionally consistent over time | Temporally stable |
| Cumulative meta-analysis | Length of stay | Effect remained directionally consistent over time | Temporally stable |
| Cumulative meta-analysis | Economic outcomes | Early fluctuation; final estimate non-significant | Less stable over time |
| Egger's test (publication bias) | Time to appropriate therapy | Intercept −0.97 (SE 2.39), p=0.700 | No significant asymmetry; underpowered (k=7) |
| Egger's test (publication bias) | Length of stay | Intercept −0.45 (SE 0.45), p=0.334 | No evidence of small-study effects |
| Egger's test (publication bias) | Mortality | Intercept 0.41 (SE 0.64), p=0.536 | No evidence of small-study effects |
| Egger's test (publication bias) | Economic outcomes | Intercept 6.97 (SE 1.86), p=0.064 | Underpowered |
| Begg's test (publication bias) | Time to appropriate therapy | Tau −0.14, p=0.773 | No significant asymmetry; underpowered (k=7) |
| Begg's test (publication bias) | Length of stay | Tau −0.37, p=0.098 | No evidence of small-study effects |
| Begg's test (publication bias) | Mortality | Tau 0.04, p=0.834 | No evidence of small-study effects |
| Begg's test (publication bias) | Economic outcomes | Tau 1.00, p=0.042 | Highly unstable with k=4; descriptive only |
| Trim-and-fill analysis | Mortality | 0 studies imputed; adjusted pooled OR unchanged | No material impact of potential unpublished small-study effects |
| Contour-enhanced funnel plots | TTAT, length of stay, mortality | Broadly symmetric visual pattern across principal outcomes | Visual assessment did not suggest major asymmetry |
| Multivariable meta-regression | Length of stay | ASP β=−1.19 (95% CI −3.52 to 1.15); Impl β=0.97 (95% CI −2.36 to 4.30); model p=0.452 | No independent predictors: 0.0% of variance explained |
| Nested subgroup analyses (within ASP-intensity strata) | TTAT and length of stay | Not estimable after exclusions | No within-stratum effect modification detected |

**J. Influence Diagnostics Summary**

| **Outcome** | **Most Influential Study (Heterogeneity)** | **Most Influential Study (Pooled Effect)** | **Outlier Detected?** |
| --- | --- | --- | --- |
| Time to appropriate therapy | MacVane et al. (2016) | Gawrys et al. (2020) | No (leave-one-out effect remained significant throughout) |
| Length of stay | Banerjee et al. (2015) | Banerjee et al. (2015) | No (leave-one-out effect remained significant throughout) |
| Economic outcomes | Box et al. (2015) | Box et al. (2015) | No (studentized residuals −1.20 to 1.56) |
| Stewardship outcomes | Gawrys et al. (2020) | Gawrys et al. (2020) | No (all within thresholds) |

**K. Prediction Intervals and Trial Sequential Analysis**

| **Outcome** | **Prediction interval** | **TSA finding** | **Interpretation** |
| --- | --- | --- | --- |
| Time to appropriate therapy | −33.96 to −0.60 h | DARIS 1533; accrued n=1453; O’Brien-Fleming boundary crossed | Robust reduction; future similar studies expected to favor faster appropriate therapy |
| Length of stay | −2.29 to −0.22 d | DARIS 2462; accrued n=3037; O’Brien-Fleming boundary crossed | Robust reduction; accrued information exceeded DARIS |
| Mortality | Crossed null | DARIS 3588; accrued n=2987; neither efficacy nor harm boundary crossed | Inconclusive; evidence insufficient for mortality benefit or harm |

For TSA, overlapping study representations were handled conservatively to avoid double counting participants; therefore, the accrued TSA sample size differs from the total mortality sample size in the main meta-analysis.

Abbreviations: ASP, antimicrobial stewardship program; BCID, blood culture identification; CI, confidence interval; d, days; h, hours; H-K, Hartung-Knapp; I², heterogeneity statistic; k, number of studies; MD, mean difference; NOS, Newcastle-Ottawa Scale; OR, odds ratio; R², proportion of heterogeneity explained; SE, standard error; tau, Begg rank-correlation coefficient; ROB, risk of bias.
